# Malignant tumor cells engender second membrane-lined organelles for self-protection and tumor progression

**DOI:** 10.1073/pnas.2317141121

**Published:** 2024-01-31

**Authors:** Tingfang Yi, Gerhard Wagner

**Affiliations:** ^a^Cytocapsula Research Institute, Cambridge, MA 02142; ^b^Centiver Ltd., Cambridge, MA 02142; ^c^Department of Biological Chemistry and Molecular Pharmacology of Harvard Medical School, Boston, MA 02115

**Keywords:** cancer, cytocapsular oncocell, cytocapsular tube, cytocapsular tumor, PMCA2

## Abstract

Cancer is a leading cause of mortality in humans, but the efficacy of current treatments for many cancers is limited, as they lack unique mechanistically defined targets. Here, we show that, upon malignant transformation, aggressive oncocells generate a second membrane exterior to their plasma membrane to form cytocapsulas (CCs) and cytocapsular tubes (CCTs), which all together constitute cytocapsular oncocells with pleotropic biological functions in cancer patient tissues in vivo. Proteomic and biochemical analyses revealed that the PMCA2 calcium pump is highly up-regulated in CCs and CCTs in malignant tumors but not in normal tissues, thus identifying a unique cancer biomarker and target for cancer therapy. Cytocapsular oncocells are universally present in solid cancers and appear in hematologic cancers in immune organs. Multi-cell malignant tumors are also enveloped by protective CC membranes. These cytocapsular tumors (CTs) generate numerous CCTs that form freeways for cancer cell metastasis to both neighboring and distant destinations. Entire cytocapsular tumor networks (CTNs) dominate physical cancer metastasis pathways in cancer patients in vivo. Later, CCTs invade micro blood vessels and release cytocapsular oncocells into the blood, providing a source of circulating tumor cells. CTNs interconnect cytocapsular tumors in primary and secondary cancer niches, creating larger cytocapsular tumor network systems (CTNSs). Primary and secondary CTNSs are in turn interconnected, forming dynamic and integrated CTNSs. Thus, interconnected cytocapsular oncocells, CTNs, and CTNSs coordinate cancer progression via the integrated cytocapsular membrane systems.

## Significance

Malignant tumor progression, cancer metastasis, cancer relapse, pan-cancer drug resistance, and immune-therapy escape present unmet challenges in cancer therapy. Moreover, the mechanisms of cancer progression in vivo are still unclear. Here, we report that cancerous cells generate cytocapsulas and cytocapsular tubes lined by a second membrane outside the cytoplasmic membrane, forming protective membranous tunnel systems that shield cancer cell proliferation, metastasis, and tumor growth. Cytocapsular oncocells are shielded from and coexist with immune cells. The developmental cycle of cancer progression in vivo suggests that the 3D cytocapsular tube networks interconnect cytocapsular tumors in primary and secondary niches, engendering dynamic cytocapsular tumor network systems. This study reveals that cytocapsular membrane systems allow for membrane-enclosed and protected cancer progression in vivo.

Curing cancer is among the most urgent challenges facing human health care (1–3). Almost 10 million cancer deaths and around 19.3 million new cancer cases occurred worldwide in 2020 alone (4). Over the last decades, many aspects of cancer progression have been studied intensively (5–11). Several models have been hypothesized for tumor progression, which together propose a diverse array of possible driving factors, including genetic alterations, genomic aberrations (discordant inheritance, DNA macro-alterations), oncoprotein promotion, signaling pathways, cell plasticity, intercellular reactions, and microenvironments (12–21). However, current clinical cancer treatment outcomes suggest that the mechanisms underlying cancer development and progression in vivo have not been fully resolved (22–24).

Recently, we reported that aggressive cancer cells engender a second membrane outside the plasma membrane in vitro, named cytocapsula (CC) or cytocapsular tube (CCT) (25). Here, we present an in-depth investigation of the biology of these previously unrecognized organelles and their links to cancer progression in vivo. We identified a biomarker Ca^2+^-ATPase 2 (PMCA2, or ATP2B2) (26–29) for the CC/CCT membrane, which allowed us to reveal the lifecycle of cytocapsular oncocells both in vitro and in vivo. We report a series of previously undocumented cancer phenomena, such as prophase-cytocapsular tumors (PCTs), cytocapsular tumor (CT), nuperphase tumor (NT), acytocapsular oncocell mass-CC/CCT complex (AMCC), cytocapsulasome, cytocapsular tumor network system (CTNS), and integrated primary and secondary CTNSs. This report expands our understanding of cancer progression in vivo to include cytocapsular membrane network-shielded oncocell devolution and evolution systems.

## Results

### Identification of PMCA2 As a Biomarker for Cytocapsulas and Cytocapsular Tubes.

**Fig. 1.**
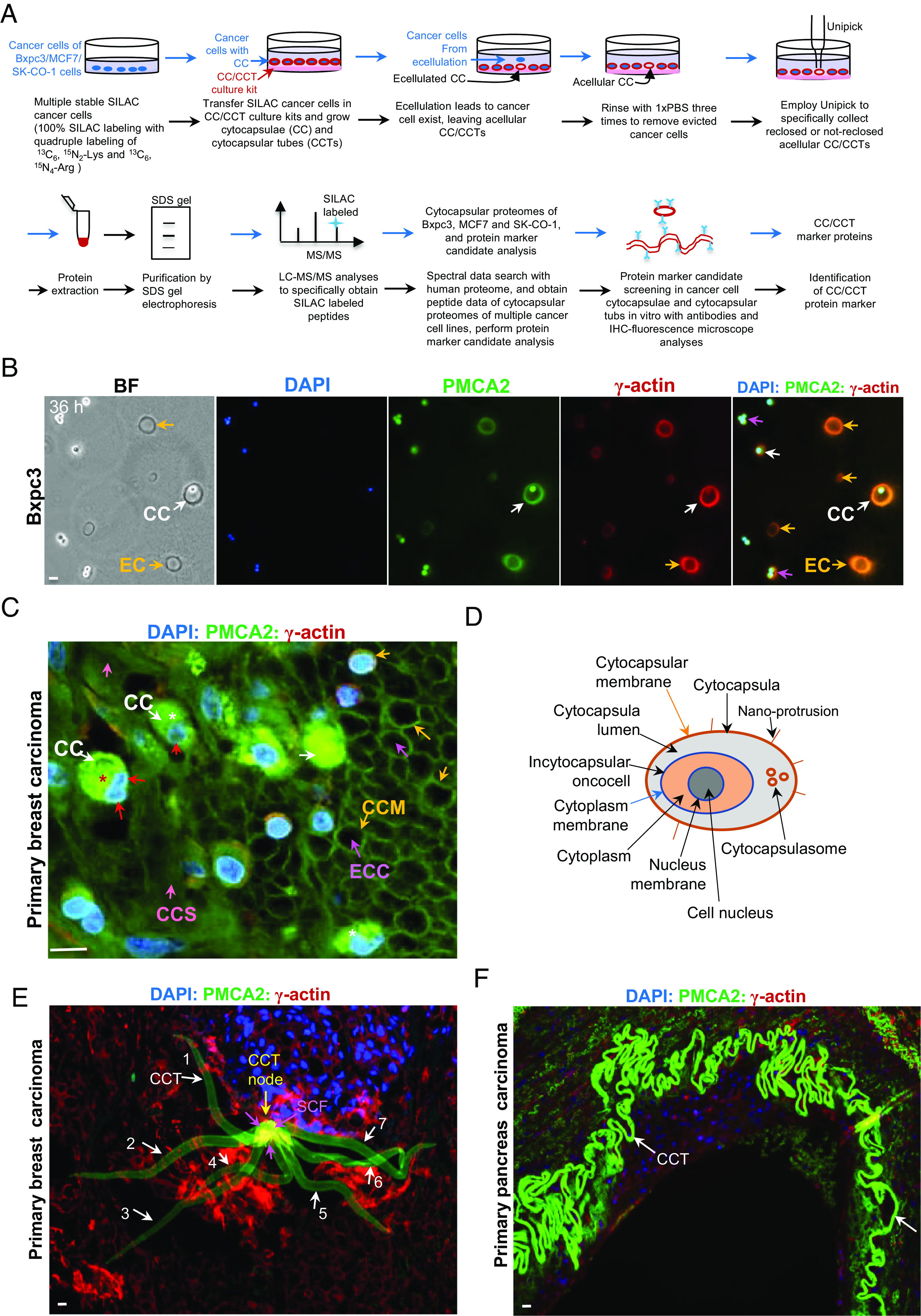
The lifecycle of cytocapsular oncocells in vitro and in human tissues in vivo. (*A*) Schematic diagram of cancer cell cytocapsular proteome analysis and cytocapsula (CC) and cytocapsular tube (CCT) molecular marker identification. (*B*) Representative immunohisto-chemistry (IHC) fluorescence microscope images of pancreas cancer Bxpc3 cells with cytocapsulas (CC) in the 3D CC/CCT culture kit matrix. Cancer cells with CC (white arrows), ecellulated cytocapsulas (EC, orange arrows), and cancer cell proliferation in CC (purple arrows) were shown. (*C*) A representative IHC fluorescence microscope image of primary breast carcinoma with cytocapsular oncocells (white arrows). Incytocapsular oncocells (red arrows), ecellulated cytocapsulas (ECC, purple arrows), cytocapsular membrane (CCM, orange arrows), and cytocapsular oncocell proliferation in CC (red asterisk) and folded CC (white asterisk) are shown. (*D*) A schematic diagram of a cytocapsular oncocell with its cytocapsula, cytocapsulasomes, and nano-protrusions. (*E*) A representative fluorescence microscope image shows that, in CCT regeneration initiation in acytocapsular oncocell mass-CC/CCT complex (AMCC) phase, a single breast cancerous cell generates multiple CCTs pointing in different directions during cancer cell’s metastasis in vivo. The seven CCTs (numbered) and multiple sectioned CCT fragments (SCF, purple arrows) are interconnected at the CCT node (yellow arrow) and exhibit a radial morphology. (*F*) A representative fluorescence microscope image of a single pancreas cancer cell generates a long, highly curved CCT in primary pancreas carcinoma tissues. (Scale bar, 10 μm.)

To explore the structure and proteins of the previously discovered cytocapsula (CC) membranes, we developed conditions for the ecellulation of cancer cells from the cytocapsulas, in order to obtain the proteome of the acellular cytocapsulas. Using CC/CCT culture kits and Unipick, we cultured and collected acellular SILAC labeled cytocapsulas (CCs) of Bxpc3 pancreas cancer cells, MCF-7 breast cancer cells, and SK-CO-1 colon cancer cells for CC proteome assays (Fig. 1*A*). A most prominent protein is the calcium pump plasma membrane Ca^2+^-ATPase 2 (PMCA2) that consistently appears in the plasma membranes of the enclosed cancer cells (Fig. 1*B*). However, PMCA2 is at much higher abundance in the CC membranes encapsulating single or multiple cancer cells (number tested, n = 601) or in ecellulated CC membranes (n = 514) in vitro. No detectable PMCA2 signal is found in the CC/CCT culture kit matrix outside the CCs or CCTs (Fig. 1*B*). PMCA2 and γ-actin constantly colocalize in cancer cell plasma membranes (n = 601) and in completely ecellulated CC membranes (n = 514) in vitro (Fig. 1*B*). Consistently, PMCA2 shows high abundance and colocalizes with γ-actin in the cytocapsular tube (CCT) membranes surrounding Bxpc3 cancer cells (n = 106, *SI Appendix*, Fig. S1*A*). The same is found in the enlarged CC membranes surrounding tumorspheres (n = 546, *SI Appendix*, Fig. S1*B*) and in the ecellulated enlarged CC membranes of tumorspheres (n = 127, *SI Appendix*, Fig. S1*B*). Thus, PMCA2 may be a molecular biomarker of CCs and CCTs in vitro. Next, we analyzed human-normal, benign-tumor, and malignant-tumor (cancer) tissues with anti-PMCA2 antibodies. In clinical normal patients (n = 14 patients, 1 tissue/patient) (*SI Appendix*, Fig. S1 *C1*) or benign-tumor tissues (n = 126 patients, 1 tissue/patient) (*SI Appendix*, Fig. S1 *C2* and Dataset S1), there are very low PMCA2 signals and no CC/CCTs, indicating that normal tissues and benign-tumor tissues don’t generate CCs/CCTs and that PMCA2 expression is tightly controlled and maintained at low abundance in both normal human tissues and benign tumors (*SI Appendix*, Fig. S1 *C1* and *C2*). In contrast, in clinical breast (n = 685 patients, *SI Appendix*, Fig. S1 *C3*) and pancreas (n = 310 patients, *SI Appendix*, Fig. S1 *C4*) carcinoma tissues, there are many long and curved CCTs with a high abundance of PMCA2 in the CCT membranes (*SI Appendix*, Fig. S1 *C3* and *C4*). In addition, there are no PMCA2 signals in the extracellular matrix (ECM) in normal, benign, and cancer tissues in vivo (*SI Appendix*, Fig. S1*C*). Thus, PMCA2 may be a molecular marker of CCs/CCTs in vitro and in vivo.

### Lifecycle of Cytocapsular Oncocells In Vitro and in Human Cancer Tissues In Vivo.

With a CC/CCT biomarker PMCA2 in hand, we tested whether cancerous cells generate CCs in vivo. Indeed, in the early stage of breast carcinoma, there are many cancerous breast cells enclosed in CCs, which shield cancerous cells inside, and isolate/protect them from the extra-cytocapsular microenvironments (Fig. 1*C*). Some breast cancer cells ecellulate from the CCs (n = 1,023), leaving acellular CCs behind, the acellular CCs align together and form acellular CC groups (Fig. 1*C*). The CC ecellulation phenomena and acellular CCs’ morphologies in vivo (Fig. 1*C*) are consistent with those in vitro (Fig. 1*B* and *SI Appendix*, Fig. S1*B*). Importantly, cancer cells proliferate in CC lumens and migrate in CCT lumens in vitro and in vivo (Fig. 1 *B* and *C* and *SI Appendix*, Fig. S1 *A* and *B*). Thus, the cytocapsular membranes not only shelter and protect cancerous cells inside but also allow cancerous cells to execute cellular behaviors and activities in the CC/CCT lumens. We termed this previously unrecognized single cancerous cell that is enclosed in an extracellular second membrane of the CC, or CCT (14) a “cytocapsular oncocell” (Fig. 1*D*). It performs cellular activities in the cytocapsular lumen.

Next, we investigated individual cytocapsular oncocell activities and behaviors in vitro and in human cancer tissues in vivo. Single cytocapsular oncocells generate long CCTs through which they migrate in vitro. CCT membranes tightly wrap the oncocell and display bulges (*SI Appendix*, Fig. S1*A*). The stretched and contracted CCTs are 2 to 3 μm in width/diameter, while the bulged CCT fragments enlarged by oncocells inside are 5 to 10 μm in width, increasing 2.5 to five folds in width and 7.85-fold to 15.7-fold in calculated perimeter (perimeter = 3.14 × diameter in a circle). Thus, CCT membranes have potent elasticity, permitting oncocells in variable sizes to dynamically migrate inside (*SI Appendix*, Fig. S1*A*). A single breast cytocapsular oncocell can engender multiple CCTs in different directions but remain connected by a node in compact breast carcinoma in vivo (Fig. 1*E*). A single pancreas cytocapsular oncocell can generate a very long and highly curved CCT in pancreas carcinoma tissues (Fig. 1*F*). These observations strongly suggest that individual cytocapsular oncocells can engender multiple, long, elastic, and robust CCTs for membrane enclosed directed cell migration. Cytocapsular oncocells proliferate in cytocapsular lumens and grow into cytocapsular tumorspheres in vitro (Fig. 1*B* and *SI Appendix*, Fig. S1*B*) and initiate tumor formation in vivo (Fig. 1*C*). Sometimes, cytocapsular oncocell ecellulation generates acytocapsular oncocells and acellular cytocapsulas followed by autodegradation into cytocapsular strands (Fig. 1*C*). In short, the lifecycle of cytocapsular oncocells includes three possible successive processes: 1) Incytocapsular oncocells proliferate and grow into cytocapsular tumors wrapped in enlarged cytocapsulas, 2) cytocapsulas elongate and generate cytocapsular tubes with oncocells migrating inside, 3) and cytocapsular ecellulation engenders acytocapsular oncocells and acellular cytocapsulas (CCs), followed by CC degradation (Fig. 1 and *SI Appendix*, Fig. S1*D*).

### Progression and Lifecycle of Cytocapsular Tumors.

**Fig. 2.**
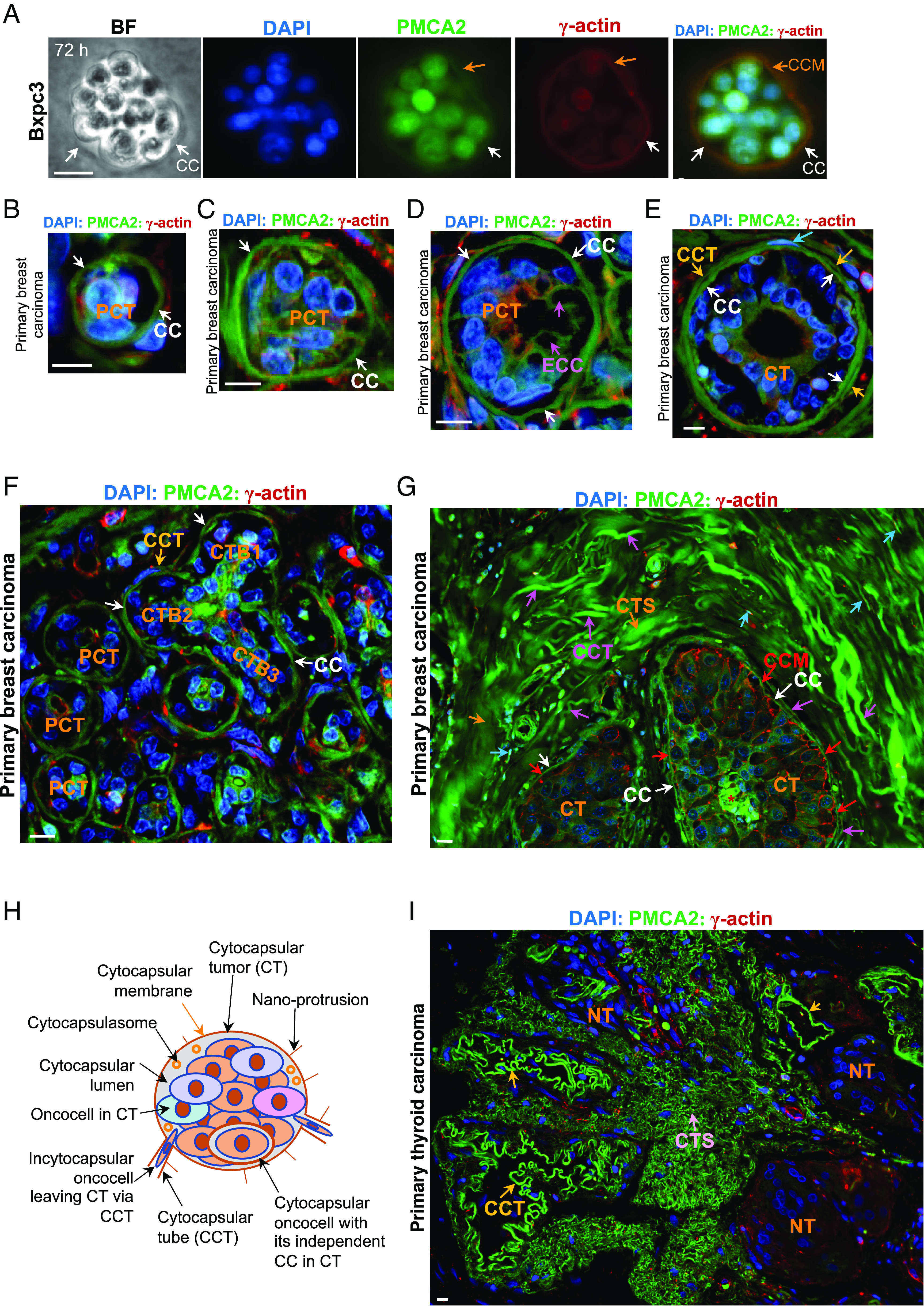
The lifecycle of cytocapsular tumors in human tissues. (*A*) Representative bright field (BF) and fluorescence microscope images of cytocapsular tumorspheres of Bxpc3 pancreas cancer cells in CC/CCT culture kit. Cytocapsula (white arrow) and cytocapsular membrane (orange arrows) are shown. (*B*) Representative IHC fluorescence microscope image of initiation-stage prophase cytocapsular tumor (PCT, <20 μm in diameter/width) in early breast carcinoma. (*C* and *D*) Representative IHC fluorescence microscope images of PCTs in early-development (*C*, <30 μm in diameter/width) and middle-development stage (*D*, <40 μm in diameter/width) stages. Cytocapsula (CC, white arrows) and ecellulated CC (ECC, purple arrows) are shown. (*E*) Early stage cytocapsular tumor (CT) with cytocapsular tube (CCT, orange arrow) extended from CTs. (*F*) Representative IHC fluorescence microscope image of breast carcinoma tissues with high PCT and CT density. Multiple CTs merge into a bigger CT. A CT with three CT branches (CTBs), cytocapsula (CC, white arrow) and cytocapsular tube (CCT, orange arrow) are shown. (*G*) Representative IHC fluorescence microscope image of developed cytocapsular tumors (CTs) with thick CCT layers wrapping the CTs outside. Cytocapsula (CC, white arrow) of CTs, cytocapsular membrane (CCM, red arrow), cytocapsular tube (CCT, purple arrows), incytocapsular oncocells (cyan arrows), and CCT strand (CTS, orange arrows) are shown. (*H*) A schematic diagram of a cytocapsular tumor with its CCTs, nano-protrusion, and incytocapsular oncocells with/without their independent CCs. (*I*) Representative image of an acytocapsular oncocell mass-CC/CCT complex (AMCC). Nuperphase tumor (NT), CCT (orange arrow), and degraded CCT strands (CTS, pink arrow) are shown. (Scale bar, 10 μm.)

Following up, we investigated whether, and if so, how individual cytocapsular oncocells grow into tumors. Indeed, at 48 h and 72 h, in the CC/CCT culture kit matrix, cytocapsular Bxpc3 oncocells proliferate in CCs and grow into tumorspheres in vitro; then, small CCs develop into enlarged CCs of increased volume, enclosing tumorspheres (n = 458, Fig. 2*A* and *SI Appendix*, Fig. S1*B*). We found that the width/diameter of CCs can increase from 8 to 10 μm in single cytocapsular oncocells to up to 40 μm in big tumorspheres in vitro, increasing up to fivefold in width/diameter. Thus, incytocapsular oncocells in CCs can support/drive small CCs in growing into large CCs, allowing oncocell proliferation and formation of large tumorspheres (Fig. 2*A* and *SI Appendix*, Fig. S1*B*). Similarly, to single cytocapsular oncocell ecellulation, some tumorspheres in large CCs undergo spontaneous ecellulation (n = 23 tumorspheres, *SI Appendix*, Fig. S1*B*). Individual oncocell ecellulation of tumorspheres in enlarged CCs generates large, reclosed and reunited, acellular, deflated, and concave CC discs (n = 35, *SI Appendix*, Fig. S1*B*). Ecellulation of tumorspheres in large CCs engender large, not-reclosed/not-reunited, acellular, deflated CCs with large open holes (n = 74, *SI Appendix*, Fig. S1*B*). These observations are evidence that solid cytocapsular oncocells grow into tumorspheres in enlarged CCs in vitro.

Subsequently, we investigated how cytocapsular oncocells progress into malignant tumors in human tissues in vivo. Initially, in the early-stage clinical breast cancer tissues examined, single cytocapsular oncocells’ extracellular cytocapsulas grew slightly larger than 15 μm in diameter/width. Single breast incytocapsular oncocells proliferate and form small oncocell masses composed of several oncocells (n ≥ 2 oncocells) in the CC lumens (n = 675, Fig. 2*B*). There is no CCT emerging from these small cytocapsular tumors at the early stage (n = 675, Fig. 2*B*). This single cytocapsular tumor with no CCTs outside in vivo is named a “prophase cytocapsular tumor (PCT).” Subsequently, cytocapsulas of PCTs develop further and increase in diameter/width, breast incytocapsular oncocells continue to proliferate and form bigger and more compact breast incytocapsular oncocell masses enclosed in the enlarged CCs (n = 312, Fig. 2*C*). The width/diameter of PCTs in the examined breast carcinoma (n = 25) ranges from 15 μm to 50 μm, and the calculated PCTs’ CC sizes (estimated in sphere morphology) range from 1,433 μm^3^ to 49,063 μm^3^ in volume (n = 89, Fig. 2 *B–D*). Sometimes, acellular cytocapsulas (n = 161) appear in breast PCT lumens (Fig. 2*D*), indicating that PCT incytocapsular oncocells generate secondary independent cytocapsulas, and these secondary cytocapsular oncocells can perform ecellulation and create ecellulated cytocapsulas. Subsequently, these ecellulated cytocapsulas in PCTs autodegrade into thin strands, followed by decomposition and disappearance, leaving liquid filled empty cavities (n = 78, Fig. 2*D*). Breast oncocells in the enlarged CCs can extend parts of CC membranes, increase CC membrane area, deform CC membranes, and form tube-shaped CCTs providing membrane-sheltered freeways for incytocapsular oncocell dissemination outside the CCs of compact tumors. These CCT membranes are the extensions of CC membranes of PCTs in vitro (n = 65, *SI Appendix*, Fig. S2*A*) and in vivo (n = 117, Fig. 2*E*). We have termed this previously unrecognized single malignant tumor a “cytocapsular tumor, CT” (Fig. 2*E*).

Furthermore, uncontrolled breast incytocapsular-oncocell proliferation produces more oncocells, forms tube-like structures in cytocapsular tumor (CT) lumens, and grows into bigger malignant tumors in the enlarged CC lumens (n = 117, Fig. 2*E*). Frequently, at the early stage, two or more adjacent small breast PCTs, CTs, or PCTs and CTs merge into larger PCTs (or CTs) via CC membrane contact, integration, degradation, and open connection formation, and form long or irregular-shaped cytocapsular tumorspheres enclosed in bigger and longer CCs (n = 145, Fig. 2*F*). Some newly merged CTs have three or more tumor branches (n = 23, Fig. 2*F*). PCTs with quite variable sizes coexist, reflecting the heterogeneity of PCTs (Fig. 2*F*). Later, with uncontrolled oncocell proliferation, the merged CTs with tumor branches remodel and transit into spherical, oval, or irregular-shaped and compact CTs (n = 132, Fig. 2*G*). Subsequently, breast CTs generate numerous CCTs when primed for oncocell metastasis. Many long CCTs surround cytocapsular tumors (CTs) and form thick CCT layers enveloping CTs, and the measured thicknesses of the CCT layers of CTs are 22 to 510 μm (n = 132 CTs, Fig. 2*G*). CTs in primary niches interconnect via dense CCT networks and form cytocapsular tumor-network systems (CTNS, Fig. 2*G*). CTs are heterogeneous in regular or irregular morphologies, (Fig. 2*G*). The above observations suggest that cytocapsular tumors with incytocapsular oncocell masses and CCTs (Fig. 2*H*) provide two inherent physical and structural drivers for the two prime features of malignant tumors (cancers) in clinical observations, that are uncontrolled proliferation and metastasis (Fig. 2 *G* and *H*).

Subsequently, the CC membranes of CTs degrade, decompose, and disappear, leaving compact acytocapsular oncocell masses without CCs (n = 814, Fig. 2*I*). We named this stage a “nuperphase tumor” (NT), (nuper, late in Latin). It is composed of the acytocapsular oncocell masses left behind after CC degradation, leaving the mass without enlarged CCs wrapping the tumor. However, some acytocapsular oncocells in NTs regenerate many new, long, and curved CCTs (n = 1,021 NTs). NT oncocells can invade into these CCTs via alloentry and disseminate, leaving decreased oncocell density in their place (n = 25, Fig. 2*I*). NT acytocapsular oncocell masses and the newly generated CCTs form a large acytocapsular oncocell mass-CT/CCT complex (AMCC) (n = 825, Fig. 2*I*). In summary, the lifecycle of cytocapsular tumors includes four successive processes: 1) single cytocapsular oncocells generation of prophase cytocapsular tumors (PCTs) without CCTs, 2) generation of cytocapsular tumors (CTs) with many CCTs for cancer metastasis, 3) CC degradation engenders nuperphase tumors (NTs) without CC enclosing oncocell masses, and 4) some acytocapsular oncocells in NT regenerate new CCs/CCTs or new small CTs and form AMCCs (*SI Appendix*, Fig. S2*B*).

Next, we examined why CCs and CCTs were previously not recognized with conventional methods. Hematoxylin and Eosin (H&E) staining does not show CCTs in cancer tissues due to the poor staining of Eosin with CCT membranes (n = 352 patients, *SI Appendix*, Fig. S3). The antibodies recognizing clinical breast cancer cell marker proteins ER, PR, and HER-2 do not recognize CCT marker proteins in these breast cancer samples (n = 213 patients, *SI Appendix*, Fig. S4*A*). Furthermore, colon cancer cell markers MSH-2 do not show features of CCTs in colon cancers (n = 86 patients, *SI Appendix*, Fig. S4*B*). Indeed, CCs and CCTs could only be detected after we observed the generation of the second membrane outside the cell membrane in CC/CCT culture kits, managed to separate the second membranes from cancer cells, obtained the CC proteome, and compared the relative abundances of the marker from ~10,000 clinically annotated cancer/normal specimens (Fig. 1 and *SI Appendix*, Figs. S3 and S4).

### Distribution of Cytocapsular Oncocells and Tumors in Human Tissues and Organs.

**Fig. 3.**
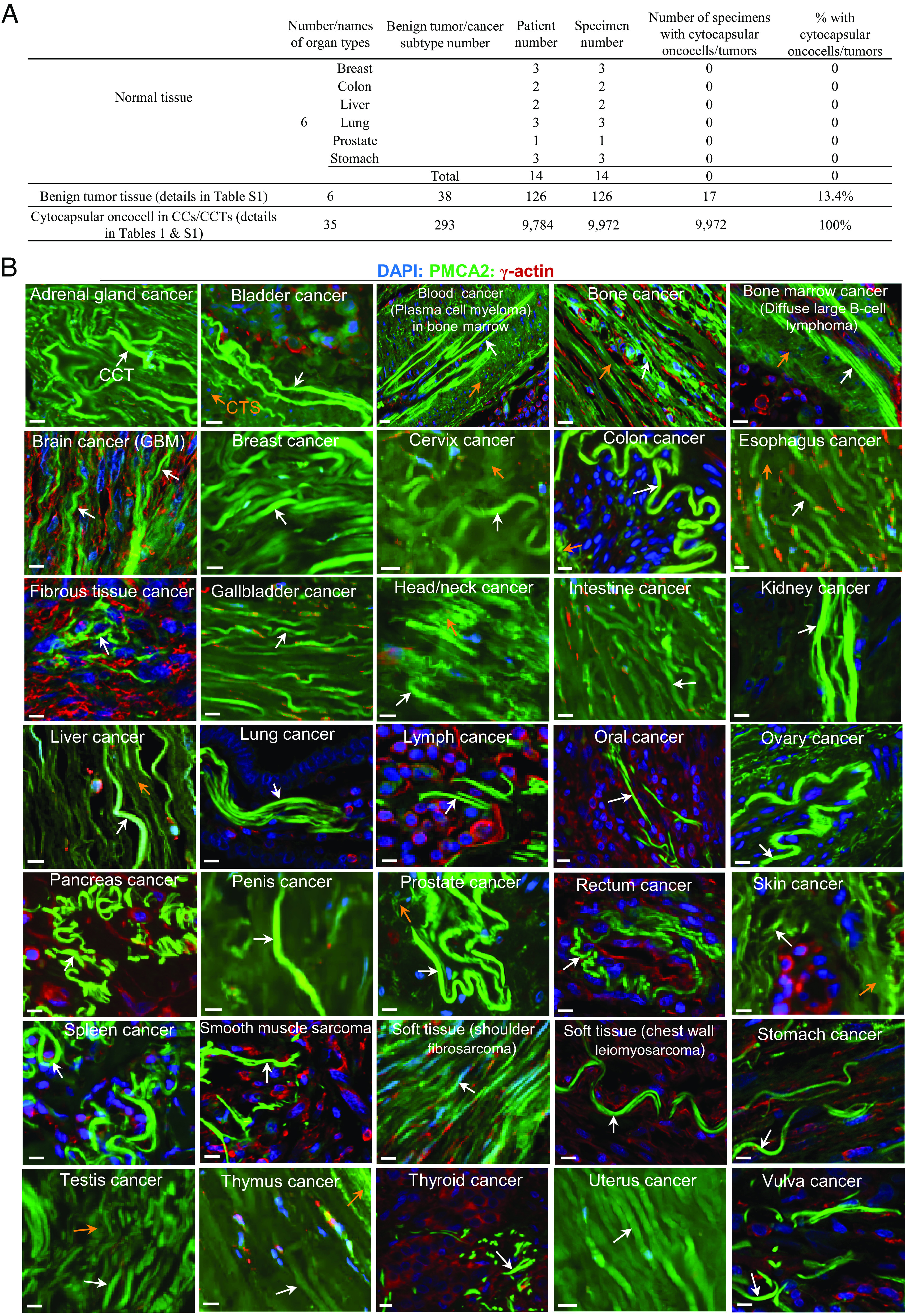
Distributions of cytocapsular-tube tumors in 35 kinds of human tissues. (*A*) Table of statistical CCT presence in the checked 35 kinds of human tissues and organs. (*B*) Representative IHC fluorescence microscope images of CCTs (white arrows) in 35 kinds of human tissues and organs. Degraded CCT strands (CTS, orange arrows) are shown. (Scale bar, 10 μm.)

Cancers universally occur in most human organs and tissues (4). Using PMCA2 as a CC/CCT biomarker, we investigated the distribution of cytocapsular oncocells and tumors in 34 kinds of human organs and tissues. In the examined six kinds of normal human organ tissues from breast, colon, liver, lung, prostate, and stomach, we don’t find detectable amounts of cytocapsular oncocells/tumors (Fig. 3*A*, n = 14 patients). In the tested 38 subtypes of benign tumors (Fig. 3*A* and Dataset S1, n = 126 patients), most benign tumors do not exhibit cytocapsular oncocells/tumors, while 13.4% of benign tumors display cytocapsular oncocells, indicating the transformation and cytocapsular oncocell occurrence in these tumors clinically annotated as benign. In the tested 290 types/subtypes of cancers of 34 kinds of human organs/tissues (except hematologic cancers in blood, patient number, n = 9,770; specimen number, n = 9,958), 100% of tested cancers show cytocapsular oncocells (Fig. 3 and *SI Appendix*, Fig. S5 and Datasets S2–S4). These observations suggest that in humans, normal and benign tumor tissues do not generate cytocapsular oncocells and that human cancers universally engender cytocapsular oncocells. Hematologic cytocapsular oncocells appear in immune organs/tissues of bone marrow, lymph node, spleen, and thymus (Fig. 3*B* and *SI Appendix*, Fig. S5 and Datasets S2–S4). There are many acytocapsular oncocells localized beyond CCs and CCTs, suggesting that acytocapsular oncocells coexist with cytocapsular oncocells in vivo. Cytocapsular oncocells exhibit three prominent features: 1) high abundance of PMCA2 in CC/CCT membranes, 2) CCTs are 3 to 10 μm in diameter/width and up to >3,000 μm in length in the sectioned specimens, and 3) localize in CCs or migrate in CCTs (Fig. 3*B* and *SI Appendix*, Fig. S5 and Datasets S2–S4). Cytocapsular oncocells’ CCTs in 283 subtypes of tested solid cancers display vast diversities, which reflects the considerable heterogeneity of cytocapsular oncocells in solid cancers (Fig. 3*B* and *SI Appendix*, Fig. S5 and Datasets S2–S4). Cytocapsular tumors universally appear in human solid cancers but not in hematologic cancers in the blood (*SI Appendix*, Fig. S5 and Datasets S2–S4). This suggests that cytocapsular oncocells are universally distributed in solid cancers and in hematologic cancers in bone marrow, lymph node, spleen, and thymus but not in normal or benign tumor tissues.

### Mechanics of Cytocapsula Growth, Generation, and Elongation and Cytocapsular Oncocell Migration in CCTs.

To get better insights, we investigated the mechanics underlying the growth of cultured cytocapsulas around oncocells in vitro. Using inverted bright field microscopy, we recorded movies of Bxpc3 tumorspheres in large CCs with wide gaps of lumen between the tumorsphere edge and CC membrane. There are multiple spike-like structures of still unknown composition (0.2 to 0.9 μm in diameter/width, 4 to 9 μm in length) in the CC lumen (named cytocapsular spikes, CS, *SI Appendix*, Fig. S6 and Movie S1). The cytocapsular spikes (checked CCs, n = 26; average: n = 4 to 10 CSs per cytocapsular tumor) interconnect the tumorsphere surfaces with the inside face of the CC membrane. They point in multiple directions, support the enlarged CC membranes, and maintain CC spherical/irregular morphologies possibly to prevent collapse (*SI Appendix*, Fig. S6 and Movie S1).

Furthermore, we observed numerous tiny (0.1 to 0.5 μm in diameter/width), spherical, membrane-enclosed particles (subsequently identified as vesicles containing PMCA2) that attach to and cover the tumorsphere surface (n = 12 cytocapsular tumorspheres, *SI Appendix*, Fig. S6). These vesicles detach from the surface of the oncocell and move freely in the cytocapsular lumen fluid (*SI Appendix*, Figs. S6 and S7 and Movies S1 and S2). Eventually, these vesicles contact, fuse to, and integrate into CC membranes, increasing CC membrane area and size, and thus promoting CC growth (*SI Appendix*, Figs. S6 and S7 and Movies S1 and S2).

Consistently in vivo, there are many tiny, membrane-enclosed vesicles with high PMCA2 abundance located both inside and outside of the cancer cell cytoplasm in early-stage stomach cancer tissues (*SI Appendix*, Fig. S8*A*). Subsequently, these vesicles fuse together and generate cytocapsulas enclosing oncocells, engendering cytocapsular oncocells (*SI Appendix*, Fig. S8*A*). These vesicles supporting CC generation/growth are present in vitro and in vivo (*SI Appendix*, Figs. S6–S8 and Movies S1 and S2). We named these vesicles cytocapsulasomes. They act as cytocapsular membrane-building material delivery cargoes and support CC generation and growth (*SI Appendix*, Fig. S8).

Cytocapsulasomes are initially engendered in the cytoplasm of acytocapsular transformed cells (n = 56 samples, *SI Appendix*, Fig. S8*A*). Subsequently, cytocapsulasomes are released outside the cytoplasm membranes (n = 87). These outside cytocapsulasomes fuse together and form small cytocapsular membrane fragments, which subsequently grow into cytocapsulas wrapping the whole oncocells and generate cytocapsular oncocells (n = 62 cancer samples, *SI Appendix*, Fig. S8*A* and Movies S1 and S2). The average number of detectable cytocapsulasomes (CS) per cytocapsular oncocell in stomach and breast cancers is 34 ± 8 CS/cell (n = 65) and 28 ± 7 CS/cell (n = 120) in vivo, respectively (*SI Appendix*, Fig. S8*B*). The above observations suggest that transformed acytocapsular cells initially generate cytocapsulasomes in the cytoplasm. Cytocapsulasomes are subsequently released from the cellular membrane, after which they randomly diffuse in the lumen and attach to the CC membrane. Then, cytocapsulasomes gradually merge together to form cytocapsulas, creating cytocapsular oncocells. Cytocapsulasomes function as cytocapsular membrane material cargo delivery carriers to drive cytocapsula growth (*SI Appendix*, Fig. S8).

Thus, we assessed the mechanics underlying cytocapsular oncocell CCT generation and elongation with time-lapse differential interference contrast (DIC) microscopy. Initially, single MCF-7 breast oncocells engender CCs, which tightly wrap the oncocell. With bleb-based motilities, single cytocapsula oncocells inside push CC membranes forward, deform CC membrane into tube-like morphologies, and produce short CCT fragments (*SI Appendix*, Fig. S9*A* and Movie S3). The initial CCT fragments are well anchored in the viscous CC/CCT culture kit matrix and maintain the wide tube shapes without being stretched or contracted into thin and long CCT tail shapes (*SI Appendix*, Fig. S9*A* and Movie S3). The cytocapsular oncocells constantly form many cytocapsulasomes, which merge and integrate into the front part of CC membranes. Cytocapsulasomes tightly keep contact with oncocell membranes, and increase CC membrane in areas. Cytocapsular oncocells constantly and dynamically form many transient blebs in various sizes. Blebs repeatedly protrude and retract in the CC, sense the extracytocapsular environments in many 3D directions, and choose and decide the motility directions. After cytocapsula creation, the oncocell migrates backward in the CCT it generated. Oncocell engenders several short cytocapsular spikes in the CCT lumens, which link the rear of the oncocell and CCT membranes (*SI Appendix*, Fig. S9*A*). Subsequently, when the single cytocapsular oncocells migrated backward in the CCT, it switched into a lamellipodia-based motility format (*SI Appendix*, Fig. S9*A* and Movie S3*A*). Average CCT elongation speed of MCF-7 cytocapsular breast oncocells in CC/CCT culture kit matrix is 1.25 ± 0.3 μm/min (n = 3 cells, *SI Appendix*, Fig. S9*B*).

Next, we investigated cytocapsular oncocell CCT generation and elongation in vivo. In the early stage of breast cancer (*SI Appendix*, Fig. S9*C*), there are many initial cytocapsular oncocell CCTs that have long and thin CCT tails (0.1 to 1 μm in width). At the initiation of CCT regeneration in AMCC phase (Fig. 1*E*), a single acytocapsular breast oncocell engenders multiple CCTs with thin tails (0.2 to 2 μm in width) as it tests multiple different migration directions during cancer metastasis. This indicates that the initial CCT fragments are stretched and contracted by the single cytocapsular breast oncocells as they move forward while the initial CCT fragments are not well anchored into the ECM (Fig. 1*E* and *SI Appendix*, Fig. S9*C*). Subsequently, when the CCTs are well anchored in the ECM by extended CCT nano-protrusions, in solid cancers the CCTs are consistently maintained at 3 to 6 μm in diameter/width (*SI Appendix*, Fig. S9*C*). The above in vitro and in vivo observations suggest that individual cytocapsular oncocells in CCs employ a bleb-based sensing and motility format. This implies a possible mechanism for how cytocapsulasomes support the growth of the CC membrane, which they push on and distort, thereby generating and then elongating CCTs while controlling their size and direction of growth. (*SI Appendix*, Fig. S9*D*). The initial CCT fragments frequently have long and thin CCT tails in vivo.

Consequently, we investigated the mechanics of cytocapsular oncocell migration in CCTs. Single Bxpc3 pancreas cytocapsular oncocells migrating via long CCTs (3 to 6 μm in diameter), appear squeezed by the stretched and contracted CCT membranes and molded into long, thin, spindle-shaped morphologies. Single cytocapsular oncocells usually appear in a single-cell mesenchymal migration format in CCTs (*SI Appendix*, Fig. S9*E*). Furthermore, using time-lapse DIC technologies, we investigated the dynamic cell migration activities in CCTs by observing multiple MCF-7 breast cytocapsular oncocells migrating in a long CCT. In most instances, multiple cytocapsular oncocells in CCTs are in single epithelial migration format, not in collective migration mode. The polarized, long thin cytocapsular oncocells in CCTs migrate forward with periodic protrusion and retraction of the leading lamellipodia and movement of the blunt cell rear. Sometimes, the long lamellipodia are completely retracted and the cells transiently appear in spherical morphologies in CCTs. CCT membranes always tightly wrap and adhere to cancer cell cytoplasm membranes and dynamically increase/decrease the CCT diameter/width locally, displaying considerable CCT membrane elasticity (*SI Appendix*, Fig. S9*F* and Movie S4). The polarized cytocapsular oncocells in CCTs can freely switch migration direction and can thus migrate bi-directionally in CCTs. Here, multiple cytocapsular oncocells freely and bi-directionally migrate through a long CCT, exhibiting dynamic cellular morphologies. The elastic CCT membranes shelter oncocells from obstacles in the heterogeneous extracytocapsular matrix outside and provide membrane-enclosed and protected freeways for cytocapsular oncocell migration (*SI Appendix*, Fig. S9 *E* and *F* and Movie S4). The average cell migration speed of multiple single Bxpc3 pancreas cytocapsular oncocells in CCT in vitro is 2.7 ± 0.5 μm/min (n = 10 cells (*SI Appendix*, Fig. S8*G*). Consistently, in the long (straight or curved) cytocapsular colon oncocell CCTs (3 to 6 μm in diameter) found in colon carcinoma tissues, cytocapsular colon cancer cells migrating in CCTs usually adopt single, long, thin, and spindle-shaped morphologies in vivo (CCT, n = 256; tissues, n = 34; *SI Appendix*, Fig. S9*H*). This indicates that cytocapsular oncocells in migration in CCTs in vivo use a single mesenchymal migration format (*SI Appendix*, Fig. S9*H*). The above observations suggest that cytocapsular oncocells can freely, dynamically, and bi-directionally migrate in membrane-enclosed and protected CCTs in a single mesenchymal migration format and long thin morphologies in vitro and in vivo. A CCT with multiple or numerous oncocells inside is topologically and bio-functionally a long and tube-shaped cytocapsular tumor and is thus termed a “cytocapsular tube tumor” (*SI Appendix*, Fig. S9 *H* and *I*).

### Primary Cytocapsular Tumor Network System Progression.

Primary malignant tumors are the origin of metastatic (secondary) cancers. Thus, we asked how primary cytocapsular tumors progress in primary niches. We assessed cytocapsular tumorsphere metastasis and secondary cytocapsular tumorsphere growth in vitro. In the CC/CCT culture kit matrix, at 68 h, single Bxpc3 cytocapsular oncocells proliferate in CCs and grow into big primary cytocapsular tumorspheres (black asterisks in, *SI Appendix*, Fig. S10*A*). Two or more primary CTs merge together via CC membrane contact, contact-interface degradation, and open connection formation, creating long and irregularly shaped CTs enclosed in long and big CCs (*SI Appendix*, Fig. S10*A*). Cytocapsular oncocells in enlarged CCs push against CC membranes and generate and elongate CCTs. CCTs interconnect and form CCT networks (*SI Appendix*, Fig. S10*A*). The CCT networks interconnect all primary and secondary CTs and form a cytocapsular tumorsphere network system (CTNS) in vitro (*SI Appendix*, Fig. S10*A*). Acytocapsular oncocells can enter CCTs via alloentry. Incytocapsular oncocells perform spontaneous ecellulation and become acytocapsular oncocells outside of the existing CCs/CCTs (*SI Appendix*, Fig. S10*A*). The ecellulated acytocapsular oncocells can generate new cytocapsulas and CCTs (*SI Appendix*, Fig. S10*B*). Incytocapsular oncocells metastasize via CCT networks (*SI Appendix*, Fig. S10*B*). In addition, cytocapsular oncocells metastasize and reside in CCT interconnection nodes, proliferate, and grow into secondary cytocapsular tumorspheres, which are integrated into the established CTNSs (n = 38 examined secondary cytocapsular tumorspheres, *SI Appendix*, Fig. S10 *A* and *B*). Incytocapsular oncocells migrate and translocate in CTNSs via CCT networks (*SI Appendix*, Fig. S10*B*). Cytocapsular tumorspheres’ spontaneous ecellulation produces acellular CT cytocapsular parts and acellular CCT fragments, making the open CCT connections between CTs visible (*SI Appendix*, Fig. S10*B*). At 78 h, all the primary and secondary CTs in a well of six-well plates are interconnected and covered by the integrated cytocapsular membrane systems, which are composed of cytocapsular tumorsphere CCs and CCT networks (CTNS number, n = 55, *SI Appendix*, Fig. S10*C*). These observations suggested that 1) primary CTs metastasize, generate CCTs, CCT networks and primary CTNSs; 2) metastasis of cytocapsular oncocells develop into secondary cytocapsular tumorspheres, and form secondary CTNSs; 3) ecellulation and alloentry allow oncocells to bidirectionally leave and enter into CCs/CCTs; and 4) primary and secondary CTNSs integrate via CCT networks and form a dynamic and integrated primary and secondary CTNS enclosed in cytocapsular membrane systems in vitro.

To better understand cancer progress, we investigated primary cytocapsular tumor network systems (CTNSs) in vivo, following subsequent development stages as outlined in *SI Appendix*, Fig. S2*B*.

#### Prophase CT (PCT) formation.

**Fig. 4.**
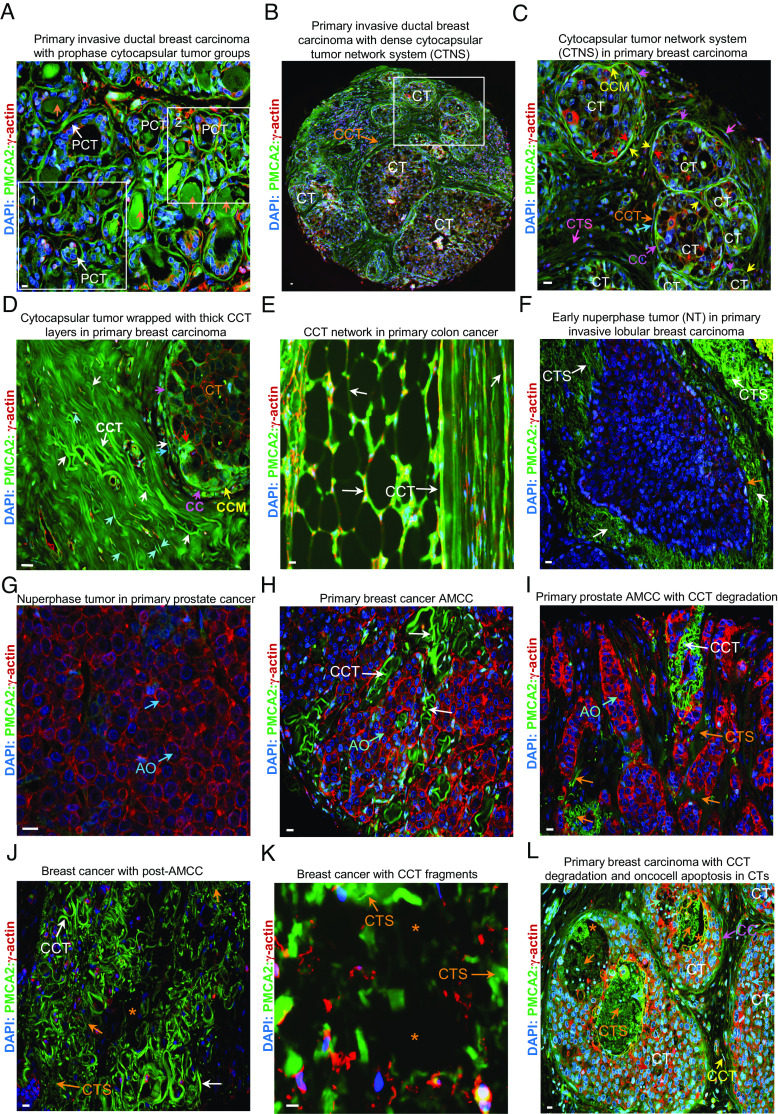
Lifecycle of primary cytocapsular tumor-network systems in vivo. (*A*) Representative image of dense prophase cytocapsular tumor (PCT, white arrows) groups in primary invasive ductal breast carcinoma. Degradation of acellular cytocapsula (CC) leads to cloud-like CC strand masses (orange arrows). Framed areas 1 and 2 are enlarged and shown in *SI Appendix*, Fig. S10 *A* and *B*. (*B*) Representative image of primary invasive ductal breast carcinoma with dense cytocapsular tumors (CTs) and CT network systems (CTNSs). The white dashed line framed area is enlarged and shown in (*C*). (*C*) Enlarged framed area of panel (*B*). CTs, cytocapsula (CC, purple arrows), cytocapsular membrane (CCM, orange arrows), CCTs (orange arrows), and degraded CCT strands (CTSs, pink arrows) are shown. (*D*) Representative image of a CT is wrapped by thick CCT layer outside. CCT outside CC (white arrows), CCT inside CC (red arrows), CC (purple arrows), and CC membrane (CCM, orange arrows) are shown. (*E*) Representative image of CCT (white arrows) networks in primary CTNSs in primary colon cancer. (*F*) Early nuperphase tumor. The CT cytocapsula is degraded with CC fragments (orange arrow) remaining. CCTs degrade into CCT strands (CTSs, white arrows). (*G*) Late stage of nuperphase tumor. There are dense acytocapsular oncocells (AO, cyan arrows) without CCTs. (*H*) Representative image of acytocapsular oncocell mass-CC/CCT complex (AMCC) in primary breast cancer. Many new CCTs (white arrows) are regenerated by some acytocapsular oncocells. (*I*) CCT degradation in AMCC. Acytocapsular oncocells (AO, cyan arrows) and CCT (white arrows) are shown. (*J*) Many acytocapsular oncocells invade into CCTs (white arrows) via alloentry, metastasize, and leave away. The local oncocell density is very low. Many CCTs are in degradation into CCT strands (CTS, orange arrow). (*K*) Severe CCT degradation with many CCT fragments and strands (CTS, orange arrow). (*L*) CCT degradation and oncocell apoptosis in cytocapsular tumor lumens. Cytocapsula (CC, purple arrows), CCT (yellow arrows), cytocapsular tube strand (CTS, orange arrows), apoptotic oncocells in CC (cyan arrows) are shown. Normal tissue cell apoptosis, acytocapsular oncocell metastasis via CCTs or apoptosis, and CCT degradation caused cavities (black areas, tissue liquefaction, orange asterisks) in panels (*J*–*L*) are shown. (Scale bar, 10 μm.)

In early-stage primary breast cancers, there are many spherical or irregularly-shaped prophase cytocapsular tumors (PCTs) of variable sizes (25 to 120 μm in diameter/width, Figs. 2*F* and 4*A* and *SI Appendix*, Fig. S11 *A* and *B*). The average prophase cytocapsular tumor (PCT) densities of early-stage breast, colon, and prostate cancers are 202 ± 59 PCTs/mm^2^, 125 ± 32 PCTs/mm^2^, and 173 ± 26 PCTs/mm^2^, respectively (specimens, n = 3 to 6, one specimen/patient, one to two subtypes/cancer type, *SI Appendix*, Fig. S11*C*).

#### PCT to CT.

Subsequently, the PCTs develop into spherical or irregular-shaped cytocapsular tumors (CTs) of variable sizes (50 to 320 μm in diameter/width, Fig. 4 *B* and *C*). The average CT densities of early-stage cancers in breast, colon, and prostate are 176 ± 38 CTs/mm^2^, 87 ± 34 CTs/mm^2^, and 158 ± 28 CTs/mm^2^, respectively (specimens, n = 5 to 12, one specimen/patient, three to four subtypes/cancer type) (*SI Appendix*, Fig. S11*D*). These data indicate that PCT and CT densities in early primary solid cancers are both statistically high. Consistent with cytocapsular tumorsphere merge in vitro, two or more small PCTs/CTs in vivo can merge into middle or large-sized PCTs/CTs (Figs. 2*F* and 4*A*). The CTs >50 μm in diameter/width start to generate a few cytocapsular tubes (CCTs). Cytocapsular tumors >70 μm in diameter/width generate many CCTs and form thick surrounding CCT layers that wrap around cytocapsular tumors (Figs. 2*G* and 4*D*). The dense CCT layers vary in thickness (30 to 803 μm) (Fig. 4*D*). Straight, curved, or coiled CCTs interconnect intensively to form 3D CCT networks, which broadly interconnect primary CTs in primary cancer niches (Fig. 4*E* and *SI Appendix*, Fig. S11 *E* and *F*). These observations suggest that cytocapsular tumors in primary niches are physically interconnected via 3D CCT networks and form primary cytocapsular tumor network systems (CTNSs) in vivo.

#### Nuperphase tumors.

While NTs grow (>800 μm in diameter/width), CCs and CCTs degrade into strands which then disappear, resulting in acytocapsular malignant tumors/oncocell masses with high cell density and lacking CCs or CCTs (Fig. 4 *F* and *G*).

#### AMCC.

The acytocapsular status of these large malignant tumors/oncocell masses is transient, resulting in a complex mix of dense oncocell masses and CCT networks not enclosed in a large CC (Fig. 4*H*).

#### AMCC with CCT degradation.

Subsequently, CCTs degrade and disappear, leaving CCT mass cavities filled with intercellular fluids (Fig. 4*I*). Meanwhile, some acytocapsular oncocells regenerate new CCTs (Fig. 4*I*). CCT degradation and regeneration coexist in the Acytocapsular oncocell Mass-CCT network Complex (AMCC) in vivo (Fig. 4*I*).

#### Post-AMCC.

Subsequently, after many oncocells have entered the CCTs and migrated away via CCTs, there are only a few oncocells left among many CCTs (Fig. 4*J*). CCTs then degrade, decompose, and disappear (Fig. 4*K*). On the other hand, CTNSs still remain in primary niches during late-stage cancer (Fig. 4*L*). The oncocells in some small or middle-sized CTs exhibit apoptosis, followed by CCT degradation (Fig. 4*L*). The above observations suggest that 1) primary CTs develop into primary CTNSs in vivo, 2) CC/CCT degradation, acytocapsular oncocell proliferation, and CC/CCT regeneration generate AMCC, 3) primary CTNSs are dynamic systems featuring CCT alloentry, ecellulation, CC/CCT generation, degradation, and regeneration, and the dissemination of cytocapsular oncocells via CCTs.

#### Normal tissues adjacent to the tumor (NATs).

NATs are necessary sites that CCTs must pass through if they are to expand. Indeed, there are large quantities of CCTs that aggressively invade into and grow through the NATs in one or multiple 3D directions in breast cancer tissues (*SI Appendix*, Fig. S12*A*). Many CCTs appear in the bone marrow NAT of plasma cell myeloma (*SI Appendix*, Fig. S12*B*). Even in the hard tissue NAT of trabecular bone of plasma cell myeloma, there are a few CCTs (*SI Appendix*, Fig. S12*C*). In the examined NATs of 68 subtypes of cancers, 100% of them harbor many CCTs and networks containing migrating cytocapsular oncocells (*SI Appendix*, Fig. S12*D*).

### Cytocapsular Oncocell Metastasis in Human Tissues In Vivo.

Most cancer deaths are due to metastases (22, 30). Thus, we assessed how cytocapsular oncocells in CCTs disseminate across diverse tissues and organs in vivo. Single CCTs invade into and spread in both loose (*SI Appendix*, Fig. S13*A*) and compact (*SI Appendix*, Fig. S13 *B* and *C*) tissues and also in the hard tissues of trabecular bone (*SI Appendix*, Fig. S12 *B–D*). 3D CCT networks enhance collective cytocapsular oncocell metastases in and across various kinds of tissues (Fig. 4 and *SI Appendix*, Fig. S12 *E* and *F*).

**Fig. 5.**
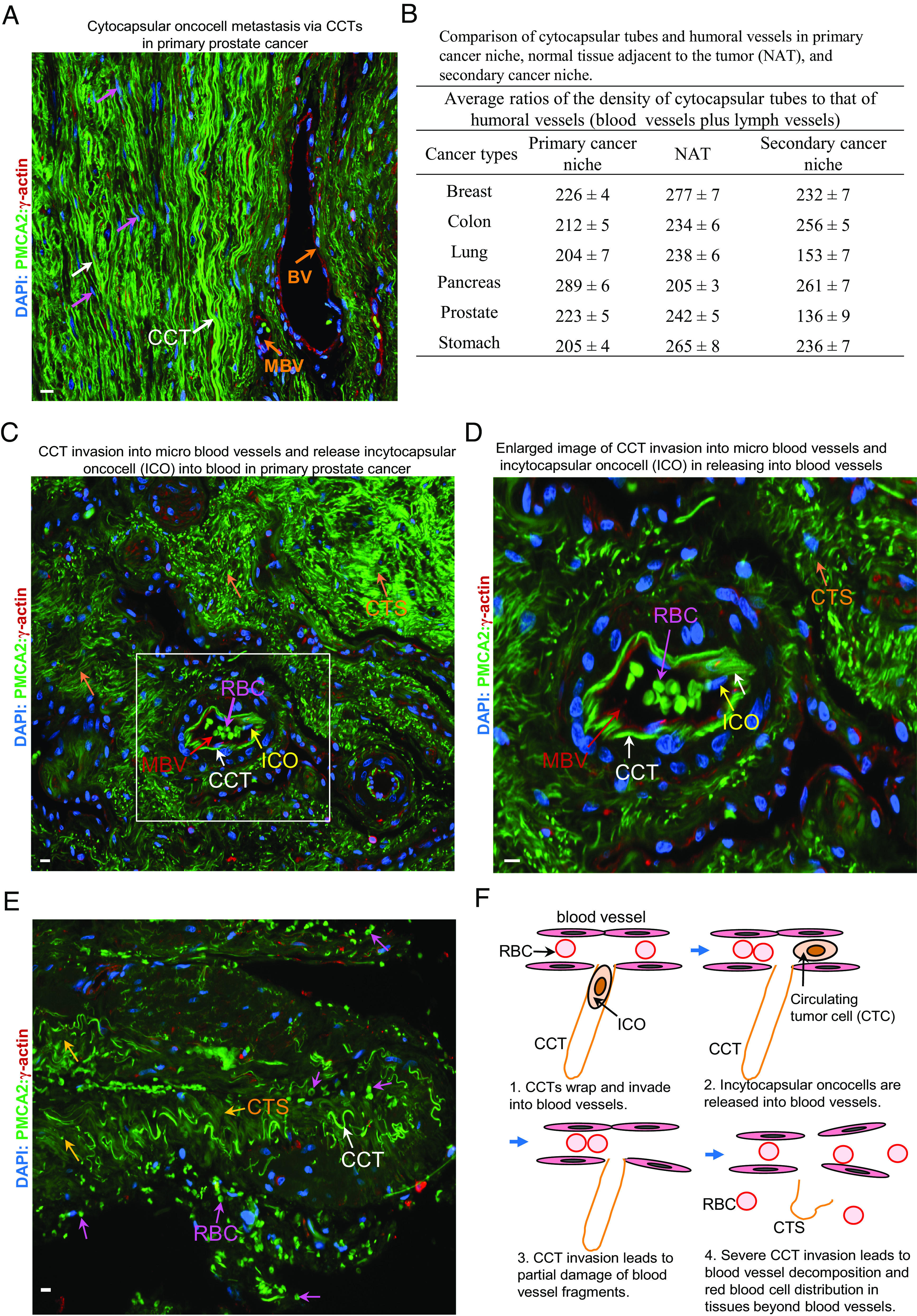
Cytocapsular tube networks dominate cancer metastasis in vivo. (*A*) Representative image of CCTs and blood vessel (BV) in primary prostate cancer tissues. CCTs (white arrows) and incytocapsular oncocell in migration in CCTs (purple arrows) are shown. (*B*) Quantitative comparison of cytocapsular tubes and humoral vessels in primary cancer niche, normal tissue adjacent to the tumor (NAT), and secondary cancer niche. (*C* and *D*) Representative image of micro blood vessels wrapped by CCTs, CCT invasion into blood vessels, and release of incytocapsular oncocells into the blood as resources of circulating tumor cells (CTCs). Framed area is enlarged and shown in (*D*). (*D*) Enlarged area from (*C*). The incytocapsular oncocell (ICO, yellow arrow) entering blood, CCT (white arrows), CCT strand (CTS, orange arrow), and red blood cell (RBS, purple arrows) are shown. (*E*) Representative image of late cancer stage with many red blood cells distributed in tissues caused by CCT invasion-damaged blood vessels. CCT (white arrows), CCT strand (CTS, orange arrow), and red blood cell (RBS, purple arrows) are shown. (*F*) Schematic diagram of CCT invasion, CTC resource, micro blood vessel damage, and red blood cell distribution in tissues beyond blood vessels. (Scale bar, 10 μm.)

Cytocapsular oncocells and CCT networks observed in the bone marrow (Fig. 3*B* and *SI Appendix*, Figs. S5 and S13*B* and Datasets S2 and S3), lymph nodes (*SI Appendix*, Fig. S5 and Datasets S2 and S3), spleen, and thymus (*SI Appendix*, Fig. S5 and Datasets S2 and S3) suggest that CC/CCT membranes can effectively shelter cytocapsular oncocells from immune cell detection and attack. CCT networks are present in 290 subtypes of cancers and in 34 types of cancerous human tissue (Fig. 3 and *SI Appendix*, Figs. S5, S12 and S13 and Datasets S2 and S3), suggesting that CCTs effectively cross all kinds of human tissues and organs for cytocapsular tumor metastasis. Large quantities of CCTs are found outside humoral vessels (Fig. 5*A* and *SI Appendix*, Fig. S14). Furthermore, in primary niches, NATs, and secondary niches, the average ratios of the density of CCTs to that of humoral vessels are up to 289-fold ± sixfold (Fig. 5*B*). The above observations suggest that cytocapsular tumor metastasis via CCT freeway systems dominates tumor metastasis in vivo. Occasionally, a very low percentage of CCTs invade into micro blood vessels and release oncocells into the circulatory systems (Fig. 5 *C–F*), indicating that humoral vessel CCT invasion-caused oncocell release is a source of circulating tumor cells in the blood. Furthermore, we tested metastases of cytocapsular oncocells in 35 types of cancers (1 to 10 secondary niches per type) and observed that cytocapsular tumors broadly disseminate to multiple secondary niches (*SI Appendix*, Fig. S5 and Dataset S5), which is consistent with clinical observations that primary tumors always metastasize into multiple tissues and organs.

### Secondary Cytocapsular Tumor Network System Progression.

Cancer metastasis and secondary tumor-caused tissue/organ biological function failure are both major causes of cancer deaths (22, 30). Thus, we assessed whether metastasized cytocapsular oncocells in secondary niches generate secondary cytocapsular tumors and CTNSs in vivo. Indeed, after breast cytocapsular oncocell CCT networks metastasize and arrive at lymph nodes, they initially form thin CCT layers which wrap around lymph nodes (*SI Appendix*, Fig. S15 *A1*). Subsequently, with CCT branching morphogenesis, more breast cytocapsular oncocell CCTs are generated and form much thicker CCT layers, enveloping the lymph nodes (*SI Appendix*, Fig. S15 *A2*). Cytocapsular oncocell CCTs of the cervix massively invade into lymph nodes with compact lymphocytes, and many metastasized-cervix cytocapsular oncocells disseminate into dense lymph nodes (*SI Appendix*, Figs. S15 *B*–*E* and S16 *A* and *B*). Breast cytocapsular oncocells in CCTs that metastasized at lymph nodes grow into large quantities of secondary breast cytocapsular tumors. They occupy the spaces of normal lymph node cells, after which many normal lymph node cells disappeared (*SI Appendix*, Fig. S15*F*). Subsequently, some oncocells in acytocapsular ovarian tumors in the omentum regenerate new CCTs CTs and CNTSs (*SI Appendix*, Fig. S15*G*). Consistently, some cervix oncocells of acytocapsular cervix tumors in the lymph node generate many CCTs in lymph nodes (*SI Appendix*, Fig. S15*H*), and rectum oncocells in acytocapsular rectum tumors in the mesentery generate large quantities of new CCTs and networks (*SI Appendix*, Figs. 15 *I* and *J* and S16 *E* and *F*). Acytocapsular oncocells invade the regenerated CCTs via alloentry and migrate away (*SI Appendix*, Fig. S15 *G*–*J*). Many secondary colon acytocapsular oncocells in the liver disseminate via regenerated colon CCTs, leaving many spaces without cells and only filled with intercellular fluids (*SI Appendix*, Fig. S15 *K* and *L*).

## Discussion

The discovery and characterization of cytocapsular tumor network systems in vivo described here and our initial report of CCs and CCTs in vitro (25) have unveiled a previously un-noticed principle of cancer. Here, we show that CCs and CCTs are universally present in cancers in vivo. This was achieved via a multi-year effort to develop procedures for cultivating CCs and CCTs in vitro at near native conditions, with the goal of separating the CC membrane from the generator cancer cell (Ecellulation) and enabling a proteomics analysis of the acellular CC membrane. As a first result, we identified the PMCA2 calcium pump as the most up-regulated factor in malignant tumors, although at very low abundance in healthy tissues. We reached this conclusion after we examined more than 10,000 samples from cancer tissue banks worldwide. PMCA2 thus emerged as the first clear and unique mechanistic target for cytocapsular tumor therapy.

Considering the fundamental new insights obtained by the discovery of the cytocapsular oncocells and tumors, we reflected on why CCs and CCTs have been undocumented until now. One primary reason is that there were no specific biomarkers for these new organelles available, and commonly used dyes don’t stain the organelles well. A great deal of time and efforts went into the technology development needed to eventually identify PMCA2 as a molecular biomarker for CC/CCTs. After the discovery of the CC/CCT membranes in in vitro and aggressive cancer cells present within the CCT culture matrix, procedures needed to be developed for the ecellulation of cancer cells from the CC second membranes to collect acellular CCs/CCTs. This in turn enabled the proteomics analysis of isolated acellular CC/CCT membranes and allowed for the comparison of the proteome of CC/CCT organelles with the proteome of healthy cells that are lacking the second extracellular membranes. *Discussion* is continued in *SI Appendix*.

## Materials and Methods

### Reagent, Antibodies, and Devices.

CC/CCT culture kits (Celldevi, https://www.celldevi.com, Cat. CD0104, six-well plates; Cat. CD0105, 12-well plates, and Cat. CD 0106, 24-well plates) and kits with glass cover slips in the well bottom and embedded by CC/CCT culture matrix layer (Cat. CD 0112, six-well plate) were ordered from Celldevi Inc. CC/CCT culture kit fixation kit (Cat. CD0201) were ordered from Celldevi Inc. Cancer cell lines of pancreas cancer cell Bxpc3, breast cancer cell MCF-7 and colon cancer cell SK-CO-1 and cell culture media were ordered from ATCC. ^13^C_6_, ^15^N_2_-L-Lysine (Cat. 88209) and ^13^C_6_, ^15^N_4_-L-Arginine (Cat. 89990) for SILAC labeling were ordered from Thermo Fisher Scientific. Unipick™ and capillary units were ordered from NeuroInDx Inc. Rabbit anti-PMCA2 antibody (polyclonal, ab3529; 1:200 dilution), mouse anti-γ-actin antibody (monoclonal, ab123034; 1:200 dilution) for immunofluorescence assay were ordered from Abcam. DAPI (1:1,000 dilution in the immunofluorescence assay) was ordered from KPL. Human normal and cancer tissue specimens described here were ordered from TissueArray and US Biolabs and were de-identified prior to use. *Methods* are continued in *SI Appendix*.

## Supplementary Material

Appendix 01 (PDF)

Dataset S01 (XLSX)

Dataset S02 (XLSX)

Dataset S03 (XLSX)

Dataset S04 (XLSX)

Dataset S05 (XLSX)

Movie S1. A Bxpc3 pancreas cytocapsular tumorsphere generates a large and spherical cytocapsula enveloping the tumorsphere. The tumorsphere engender large quantities of cytocapsulasomes (membrane-enclosed tiny particles in white color in the movie) covering the surfaces of itself. Cytocapsulasomes can detach and move into the cytocapsular lumen fluids in a random mobility format. Cytocapsulasomes can reach the enlarged cytocapsula membrane, and integrate into cytocapsula membrane, and increase the membrane area sizes of cytocapsula membranes, driving cytocapsula growth or cytocapsular tube elongation. Representative bright field microscope images are extracted and shown in **Fig. S6**.

Movie S2. A Bxpc3 pancreas cytocapsular tumorsphere generates a large and spherical cytocapsula enclosing the tumorsphere, and engender a short and big “L” shaped CCT inside the CC lumen. Two individual cytocapsular oncocell in the CC lumen generate their independent CCs and perform ecellulation, and leave two acellular CCs in the large CC lumen. The cytocapsular tumorsphere generates a lot of cytocapsulasomes, and many of them are detached, migrate and arrive at, and contact with the inner side of the enlarged CC membrane. Cytocapsular oncocell engendered cytocapsulasomes are building blocks and shuttering cargos for CC growth and CCT elongation. Representative bright field microscope images are extracted and shown in **Fig. S7**.

Movie S3. Single breast MCF-7 cancer cell in CC/CCT culture kit matrix generates a cytocapsula enclosing itself, and produces a cytocapsular oncocell. The cytocapsular oncocell employs blebbased mobility for migration forward, and engender a cytocapsular tube (CCT) behind. Subsequently, the cell switch into a lamellipodia-based motility and migrate in the CCT it generated and migrate back. Single cytocapsular oncocells can generate CCT and migrate inside. Representative DIC images are extracted and shown in **Fig. S9A**.

Movie S4. Multiple cytocapsular oncocells in a single CCT employ a single epithelial migration format, and dynamically and bi-directionally migrate in the CCT. The polarized, thin and long cytocapsular oncocells in the CCT migrate forward with periodic protrusion and retraction of the leading lamellipodia and movement of the blunt cell rear. During cancer cell migration in CCT, CCT membranes tightly adhere to oncocell cytoplasm membranes and dynamically increase/decrease the CCT diameter/width locally, displaying considerable CCT membrane elasticity. The polarized and mesenchymal cytocapsular oncocells in CCTs can freely switch the migration direction back and forth and can thus migrate bi-directionally in CCTs. Representative DIC images are extracted and shown in **Fig. S9F**.

## Data Availability

All study data are included in the article and/or supporting information.
