# Supplementary material for: Malignant tumor cells engender second membrane-lined organelles for self-protection and tumor progression
Source: Proc Natl Acad Sci U S A. 2024 Jan 31;121(6):e2317141121. doi: 10.1073/pnas.2317141121 (PMC10861905; doi:10.1073/pnas.2317141121)
Supplement: Supplementary file 1 — Appendix 01 (PDF) [file pnas.2317141121.sapp.pdf]

Supplementary Information:

Malignant tumor cells engender second membrane-lined organelles for self-protection and tumor progression

Tingfang Yi[1,2*] and Gerhard Wagner[1,2,3*]

1. Cytocapsula Research Institute, 245 First Street, Cambridge, MA, 02142 USA
2. Centiver Ltd., 245 First Street, Cambridge, MA, 02142 USA
3. Department of Biological Chemistry and Molecular Pharmacology of Harvard Medical School, 240 Longwood, Boston, MA, 02115 USA

[*]To whom correspondence may be addressed**.** E-mail:tingfang_yi@centiver.com, or Gerhard_Wagner@hms.harvard.edu.

This PDF file includes:

Continuation of Discussion
Continuation of Materials and Methods
Figures S1 to S18
Legends for figures S1 to S18
Legends for Movies S1 to S4
SI References

Other supporting materials for this manuscript include the following:

Movies S1 to S4
Tables S1 to S5

Discussion continued from main text

Considering the fundamental new insights obtained by the discovery of the cytocapsular oncocells and tumors, we reflected on why CCs and CCTs have been undocumented until now. One primary reason is that there were no specific biomarkers for these new organelles available, and commonly used dyes don't stain the organelles well. A great deal of time and efforts went into the technology development needed to eventually identify PMCA2 as a molecular biomarker for

CC/CCTs. After the discovery of the CC/CCT membranes in *in-vitro* and aggressive cancer cells present within the CCT culture matrix, procedures needed to be developed for the ecellulation of cancer cells from the CC second membranes to collect acellular CCs/CCTs. This in turn enabled the proteomics analysis of isolated acellular CC/CCT membranes and allowed for the comparison of the proteome of CC/CCT organelles with the proteome of healthy cells that are lacking the second extracellular membranes. Using these processes, PMCA2 was found to be highly upregulated in the CCT membranes when compared to cell membranes of healthy cells. Subsequently, we interrogated the upregulation of PMCA2 in ~10,000 annotated clinical samples from tissue banks worldwide, which revealed the high upregulation of the PMCA2 calcium pump in cancer specimens compared to healthy cell membranes. This enabled the science described here, and in turn reveals numerous new insights into the mechanisms of cancer.

There is still a question as to why conventional assays didn't detect CCs or CCTs. Here are a few possible reasons: For *in vivo* assays: 1) H&E dyes show poor staining of membranes of CCs/CCTs; 2) the high-abundance CC/CCT marker proteins have previously been unknown and high-quality antibodies recognizing CC/CCT marker proteins were not available; 3) the low resolution of common fluorescence microscopes, or a high autofluorescence noise background caused low signal/noise ratios, and CCs/CCTs were missed; 4) compact acytocapsular oncocell masses are emphasized and signals of CCs/CCTs are ignored, and treated as extracellular matrix (ECM) or considered as unknown phenomena not worth further study; 5) IHC-horseradish peroxidase (HRP) methods with anti-PMCA2 antibodies cannot show clear images of CC/CCT IHC images due to the low resolution caused by diffusion of colored product particles, and caused by the unfamiliar long/large, continuous, curved and bumpy membrane structures of CC/CCTs; 6) IHC-fluorescence methods involve multiple successive procedural steps, wherein even one or two

steps being carried out improperly (such as insufficient antigen/epitope exposure of highly folded CC/CCT membranes, non-specific antibody binding/aggregation, and insufficient/low quality in antibody binding specificity/affinity/sensitivity) will lead to low quality outcomes or missed CCs, CCTs, and CCT strands, even when using anti-PMCA2 antibodies. It will also lead to cancer tissue-sample compositions with only acytocapsular oncocell masses, that contain too little PMCA2, CC/CCT auto-degradation/auto-decomposition in tissue samples, and autopsy samples experiencing post-life CC/CCT degradation and decomposition.

For *in vitro* analyses: 1) ECM mimics (such as collagen gel, Matrigel matrix) in cell culture plates have difficulty achieving the precise necessary biochemical, biophysical, and biomechanical characteristics (e.g., polymerization, density, and viscoelasticity) of ECM; 2) Conventional cancer cell line-derived xenografts (CDX), patient cancer cell-derived xenografts (PDX) and orthotopic-PDX (O-PDX), which are popularly used as mimics of cancer and assays for testing cancer drug candidates, usually do not generate CCs or CCTs for reasons that currently remain unknown.

These key insights were paralleled by the discovery of numerous new aspects of cancer malignancy, which dramatically widened our understanding of cancer as a whole. The most prominent new mechanistic insights are as follows:

(i) Discoveries of the cytocapsular oncocell, cytocapsular tumor, cytocapsulasome, cytocapsular tumor network system (CTNS), integrated primary and secondary CTNSs.

(ii) All malignant tumors generate a second extracellular membrane, reminiscent of ancient forms of life, such as mitochondria, chloroplast and gram-negative bacteria. The presence of the PMCA2 calcium pump in the second extracellular membrane is essential for malignancy in all known cancers. The absence of high levels of PMCA2 defines tissues as benign and normal. This is a potent tool for cancer diagnosis and therapy.

(iii) PMCA2 has been validated as marker for malignant cancer by the FDA (1) .

(iv) PMCA2-containing cytocapsulasome vesicles appear to promote CCT elongation, contribute to tumor progression and invasion into even solid tissue or hard trabecular bone.

(v) Multicellular malignant tumors are surrounded by CC membranes.

(vi) Alloentry allows acytocapsular oncocells to invade into CCTs followed by CCT-directed metastasis; Ecellulation of large CCs creates cell-less scaffolding structures.

(vii) CCT mediated disposal of tumor cells and normal cells creates empty liquid filled holes rendering tissue non-functional (**Fig. S10**).

(viii) Initial metastases involve the transport of tumor cells via CCTs, which only later penetrate humoral vessels and thus effect cancer dissemination via previously known pathways.

(ix) Secondary cytocapsular network systems use CCTs for the dissemination of multi-cell cancer dissemination.

(x) The cytocapsular oncocell, cytocapsular tumor, cytocapsular oncocell metastasis in CCT networks, and cytocapsular tumor network systems in cancer development and progression, which may facilitate research into effective therapies against cancers.

The discovery of CCs and CCTs opens up a number of important questions that may be pursued in future research. (i) What causes the upregulation of PMCA2 and other factors in CCs/CCTs upon malignant transformation? (ii) Furthermore, what are the molecular mechanisms that cause the generation of the second extracellular membrane? Some insight may come from studies of existing or ancient biosynthesized bacteria with extracellular membranes, such as

mitochondria, chloroplasts and gram-negative bacteria. Also, high resolution recordings of transforming cells as shown in **Fig. 1** or **Movies S1-S4** may provide new insights.

The discovery of the progression of cytocapsular oncocells and CTs, CTNSs, and integrated primary and secondary CTNSs in human organs and tissues as described in this report may facilitate further cancer research into early screening, prognosis, diagnosis, drug development, therapy and treatment. Extended analysis of the proteome and metabolome of CCTs of multiple cancer types may provide additional insights into cancer mechanisms and yield new targets for cancer diagnosis and therapy (in progress). The time course and causality of cancer progression suggested here was mostly based on comparing static snap shots from a large number of tissues samples that are consistent with mechanisms of progress. However, there is a need for better monitoring cancer growth *in vivo* at the resolution reported here; this may be enabled by the tools developed here.

Understanding the mechanisms of cancer progression in human organs/tissues is critical for cancer research, early screening, prognosis, diagnosis, drug development, therapy and treatment (2-3). Here we investigated mechanisms of cancer progression in human tissues and organs *in vivo*, and found that cytocapsular oncocells, cytocapsular tumors, and cytocapsular tumor network systems drive membrane-sheltered cancer development and progression. The identification of PMCA2 as a cytocapsular membrane protein marker makes the previously invisible CCs/CCTs organelles visible. This opens a new window for research into the biological mechanisms of malignant tumors. It provides new possibilities for understanding cytocapsular oncocells/tumors with newly discovered organelles/compartments, and may open new avenues for cancer research and therapies. The new discovery and features of the cytocapsular oncocell, cytocapsular tumor, and cytocapsular tumor systems (CTNSs) unveil a long-term unrecognized

mechanism that cytocapsular membrane systems function as integrated, membrane-encompassed, and protective powerhouses that coordinate cytocapsular oncocell proliferation, cytocapsular tumor growth, cytocapsular cancer metastasis via and in CCT networks.

The discovery of the combined extracellular membranes of cytocapsular oncocells, cytocapsular tumors, and CCT networks may facilitate the research into the development of better pharmaceutical therapies which minimize membrane barrier caused pan-drug resistance and immune attack escape. Our study raises the question of what the molecular mechanisms are that underly CC/CCT initiation of transformed cells *in vivo*. The characterizations of the cytocapsular oncocell, cytocapsular tumor, and cytocapsular tumor systems (CTNSs) unveil a long-term unrecognized mechanism that cytocapsular membrane systems function as integrated, membrane-encompassed, and protective powerhouses coordinating cytocapsular oncocell proliferation, cytocapsular tumor growth, cytocapsular cancer metastasis via CCT networks. Cytocapsular oncocell metastasis in cytocapsular membrane-enclosed integrated primary and secondary CTNSs facilitates cancer metastasis in several aspects: 1) free of obstacles from heterogeneous extracytocapsular matrix and neighboring cells, 2) minimizes or eliminates extracytocapsular attacks from immune cells and stresses from toxic molecules (such as cancer drugs), 3) have membrane-enclosed physical freeway systems for safe and efficient cancer metastasis, 4) can perform bi-directional migration in the dynamic and integrated primary and secondary CTNSs.

These observations of integrated primary and secondary CTNSs may shed light on cancer research, indicating the importance of considering the primary and secondary tumors as a united, dynamic and cytocapsular membrane-encompassed system, instead of the fragmented view of

separated primary and secondary tumors as largely separate; the latter approach clinically displays transient and limited success, followed by irreversible and undruggable cancer relapse. This may facilitate research into the development of better pharmaceutical and immune therapies with less or no membrane barrier caused pan-drug resistance and immune attack escape. The CCT invasion into humoral vessels and oncocell release as a circulating tumor cell resource are consistent with clinical observations that massive tumor metastases occur while the circulating tumor cells are rare in blood cells *in vivo*. Our study raises the question of what the molecular mechanisms underlying CC/CCT initiation of transformed cells are *in vivo.*

Interestingly, sometimes, many red blood cells randomly spread in large areas in the secondary hepatocellular carcinoma in cerebrum of brain, indicting some (micro)blood vessels are broken and leaky caused by CCT invasion and (micro)blood vessel decomposition, and red blood cells are released. The above observations suggest that metastatic cytocapsular tumor progression in the secondary niches includes 6 major successive stages: 1) arrival and invasion of metastatic cytocapsular oncocells in CCTs in the secondary niches, 2) generation of secondary cytocapsular tumors, 3) formation of secondary CTNSs, 4) formation of dynamic integrated primary and secondary CTNSs via CCT networks, 5) CC/CCT degradation and formation of AMCCs, 6) generation of new CCTs for oncocells' next metastasis. These observations suggest that metastasized cytocapsular oncocells have capacities to generate large quantities of small/middle-sized secondary cytocapsular tumors and dense CTNSs in the secondary niches (in neighboring or far-distance organs/tissues), and lead to massive normal cell disappearance followed by affected, harmed, or even failed biological functions in the secondary niches related tissues/organ. In summary, our results suggest that cytocapsular oncocells, cytocapsular tumors, and integrated

primary and secondary CTNSs coordinate membrane- sheltered cancer progression in human (**Figs. S17-18**).

The discovery of the progression of cytocapsular oncocells and CTs, CTNSs, and integrated primary and secondary CTNSs in human organs/tissues described in this report may facilitate further cancer research, early screening, prognosis, diagnosis, drug development, therapy and treatment.

Open questions include: What is the metabolome of cytocapsulas in diverse cancers, how does the proteome vary between cancers? The biological functions and underlying molecular mechanisms of a PMCA2 γ-actin axis in cancer formation and metastasis need further research to explore (6).

Continuation of Materials and Method

Stable SILAC labeled CC/CCT culture and collection for CC/CCT proteome analyses

Following CC/CCT culture kit usage instructions, stable SILAC labeled cancer cells of Bxpc3, MCF-7 and SK-CO-1 with $^{13}C_6$, $^{15}N_2$-L-Lysine and $^{13}C_6$, $^{15}N_4$-L-Arginine were implanted in CC/CCT culture kits (6-well plates) with cell culture media with $^{13}C_6$, $^{15}N_2$-L-Lysine and $^{13}C_6$, $^{15}N_4$-L-Arginine (7,8). Stable SILAC labeled cancer cells generate stable SILAC labeled cytocapsulas (CCs) and cytocapsular tubes (CCTs). Sometimes, some incytocapsular oncocells are evicted from CCs and CCTs *in vitro*. After ecellulation, evicted cancer cells were washed away by three washes with 1xPBS. Acellular CCs/CCTs (ECC/ECCTs) were collected by Unipick and kept on ice followed by storage at -80°C. The proteome assays of acellular CCs with holes will lead to the loss of some proteins in the CC lumen, and refined methods are needed to explore the complete proteome of CCs in future.

More than 400,000 stable SILAC labeled acellular CCs/CCTs per CC/CCT proteome analysis sample were collected in > 4 years.

Cytocapsular tumorsphere and cytocapsula growth *in vitro*, immunohistochemistry staining and imaging

Pancreas cancer Bxpc3 cells were implanted in CC/CCT culture kit (Cat. CD 0112, Celldevi) following the kit manual. At 36h, Bxpc3 cells generated cytocapsulas (CCs). Some cytocapsular oncocells performed ecellulation. Cytocapsular oncocells and ecellulated CCs were performed fixation kit and immunohistochemistry staining. At different time of 48h, 72h, 68h, 74h, 78h, 84h, 96h, 108h after cell implantation, Bxpc3 cancer cells engender cytocapsular oncocells, and grow into cytocapsular tumorspheres in different sizes with CC tightly wrapping oncocell mass or with wide cytocapsular lumens, and ecellulation of cytocapsular tumorspheres. These cytocapsular tumorspheres and ecellulated cytocapsular tumorspheres were fixed by Celldevi Inc. CC/CCT fixation kit (Celldevi, CD0201) in the 6-well plate, and then taken out and put onto slides, followed by immunohistochemistry (IHC) staining.

Liquid chromatography tandem mass spectrometry CC/CCT proteome analyses

The proteins in collected ECCs/CCTs with stable SILAC labeled with $^{13}C_6$, $^{15}N_2$-L-Lysine and $^{13}C_6$, $^{15}N_4$-L-Arginine were extracted and purified by SDS-gel electrophoresis. After Coomassie blue staining, the SDS-gel strip of one sample was cut into 4-5 gel slices. After in-gel digestion12.5 ng/μL trypsin, the digested peptides were extracted and enriched. The enriched peptides were used for liquid chromatography tandem mass spectrometry (LC-MS/MS) analyses as previously described (8). The enriched peptide fractions were analyzed by liquid chromatography tandem mass spectrometry (LC-MS/MS) on an LTQ Orbitrap Velos mass

spectrometer (Thermo Scientific) equipped with a Thermo Fisher Scientific nanospray source, an Agilent 1100 Series binary HPLC pump, and a Famos autosampler. Peptides were separated on a 0.125 × 180 mm fused silica microcapillary column with an in needle tip (made in-house) with a ~5-µm i.d. The silica microcapillary column was packed with magicC18AQ C18 reverse-phase resin (5-µm particle size, 200-Å pore size; Michrom Bioresources). Separation was performed by applying a 57-min gradient from 7% to 28% acetonitrile in 0.125% formic acid. The mass spectrometer was operated with default settings: full MS [automatic gain control (AGC), 1×10^6; resolution, 6×10^4; m/z range, 375–1,800; maximum ion time, 1,000 ms]; MS/MS (AGC, 5×10^3; maximum ion time, 120 ms; minimum signal threshold, 4×10^3; dynamic exclusion time setting, 30 s; charged ions and ions for which no charge state could be determined were excluded MS/ MS selection). Triplicated independent experiments were performed.

Database Searches, Data Filtering, Validation of Protein Detection Rate, and Proteome Analyses.

The spectral data were searched with SEQUEST[2] against a database containing the human protein sequence database (www.ensembl.org/index.html) together with the reversed complement. The LC-MS/MS identifications were filtered to 0.98% protein false discovery rate (FDR) and 0.1% peptide FDR. The peptide quantification and phosphorylation site localization were analyzed using in-house software and Ascore as previously described (8).

Cytocapsular tumorsphere and cytocapsula growth *in vitro*, immunohistochemistry staining and imaging

IHC staining was performed with rabbit anti-PMCA2 polyclonal primary antibodies (1:200 dilution), mouse anti-γ-actin monoclonal primary antibodies (1:200 dilution), Goat anti-Mouse IgG (H+L) Highly Cross-Adsorbed Secondary Antibody, Alexa Fluor Plus 555 (Thermo Fisher), and Goat anti-Rabbit IgG (H+L) Highly Cross-Adsorbed Secondary Antibody, Alexa Fluor Plus 488, Thermo Fisher), and DAPI staining (1:000 dilution). Fluorescence images were taken with a Nikon 80i upright microscope with a 20× or 40× lens. All images were obtained using MetaMorph image acquisition software and were analyzed with ImageJ software.

CCT Histology and Immunohistochemical Staining Analysis

The 9972 formalin-fixed, paraffin-embedded (FFPE) human cancer tissue specimens (4-5μm in thickness) from 9784 cancer patients, 14 human normal tissue FFPE specimens form 14 patients, and 126 human benign tumor tissue FFPE specimens from 126 patients were processed immunohistochemistry and hematoxylin and eosin (H&E) staining. Immunohistochemical fluorescence tests were performed to stain cytocapsular tubes using rabbit anti-PMCA2 polyclonal primary antibodies (1:200 dilution), mouse anti-γ-actin monoclonal primary antibodies (1:200 dilution), Goat anti-Mouse IgG (H+L) Highly Cross-Adsorbed Secondary Antibody, Alexa Fluor Plus 555 (Thermo Fisher), and Goat anti-Rabbit IgG (H+L) Highly Cross-Adsorbed Secondary Antibody, Alexa Fluor Plus 488, Thermo Fisher), and DAPI staining (1:000 dilution). Fluorescence images were taken with a Nikon 80i upright microscope with a 20× or 40× lens. All images were obtained using MetaMorph image acquisition software and were analyzed with ImageJ software.

Time-Lapse DIC Microscopy and Videos.

Time-lapse DIC microscopy analyses of cytocapsula elongation and cell migration were performed using a Nikon Ti motorized inverted microscope and a digital Hamamatsu ORCA-ER cooled CCD camera with a 20× lens. The time-lapse microscope was equipped with DIC, phase contrast, and epi-fluorescence optics, a Prior ProScan III mo- torized stage and shutters, a perfect focus system, and an Okolab 37 °C, 5% CO2 cage microscope incubator (Okolab). Images were taken every 30 s over the course of ∼10–36 h. All images were obtained using MetaMorph software. Tracks made by 2 h of cytocapsula elongation were obtained using MetaMorph and ImageJ software. Cytocapsula elongation velocities were also calculated using length and time measurements. Movies were prepared using the images collected via time-lapse and Meta- Morph software (15 frames/s).

Bright filed microscope and videos

Bright field microscope analyses of cytocapsula growth with cytocapsulasomes activities were performed using Nikon Eclipse TS2 Inverted Routine Microscope with a DS-FI3 Microscope Camera with a phase contrast 20x lens. The videos were taken using NIS-Elements software (25fps, frame per second).

Imaging Acquisition.

DIC and fluorescence images of fixed cells (with or without cytocapsulae) were taken with an 80i upright microscope and a digital Hamamatsu ORCA-ER cooled CCD camera with a 20× or 40× lens. The bright-field phase-contrast image was taken using a Nikon digital camera. The cytocapsula initiation ratio per high-performance field (HPF; 200×) and the number of elongated cytocapsulae per high-performance field were quantified. All images were obtained using MetaMorph image acquisition software and were analyzed with ImageJ software.

Data collection

Cytocapsular tubes (CTs, not sectioned, longitudinally sectioned, and cross sectioned) without degradation (3~6μm in measured diameter) were counted using a fluorescence microscope and ImageJ. The presence of CTs degrading into thick strands (1~2μm in measured diameter), thin strands (0.2~1μm in measured diameter), or the disintegration state were reported without quantification. The patients providing formalin-fixed paraffin-embedded tissue samples gave informed consent that they understood that the biopsies (needle biopsy or surgical biopsy, from US Biomax) were performed for *in vitro* research purposes only. Comparative deidentified samples of normal tissues, benign tissues, carcinoma *in situ*, cancer, paracancer, metastatic tissues with their cancer stages identified according to the tumor (T), node (N), and metastasis (M) TNM system (cancer stages: 0, I, II, III, IV) were obtained from archival materials (Tables S3-4). The cancer, paracancer and metastatic cancer tissues, in which the original cancer niches were identified by indicated cancer specific molecular markers, were identified by hospital pathology laboratories and obtained from archival material. Autopsy tissues samples have many post-life CC/CCT degradation and CCs/CCTs will not be quantified. Biopsy samples from FFPE with fresh tissues present high fidelity of CC/CCT status and CCs/CCTs are quantified and reported.

Quantification and Statistical Analysis.

The statistical methods used for comparisons are indicated in the relevant Fig. legends and in the sections below. The diameters, widths, and lengths of cytocapsulae and cytocapsular tubes were measured with MetaMorph or ImageJ. The time of individual cytocapsulae and cytocapsular tubes was counted from cytocapsula generation to acellular cytocapsula (or cytocapsular tube) decomposition. For lifetime of cytocapsular oncocell and cytocapsular tumorsphere assays, at least 20 cytocapsular oncocell or cytocapsular tumorsphere were measured per condition, and two-tailed

Student's test was used to determine statistical significance. The movie taken time was labeled as hour : minute : second (in **Figs S6-7**), while times after cell implantation were 96h in **Fig.S6** and 108h in **Fig.7**. In **Fig.S9**, the movie taken time is just after cell implantation. The graph plots are mean ± SD. In CCT analysis in cancer types/subtypes, at least 3 samples per cancer subtype were checked (in **Figs. 3A** and **5**, **Datasets S2, S5**). In cytocapsular tube quantitation assays, for each specimen, the number of fully intact cytocapsular tubes was counted in 5 areas (0.35mm x 0.35mm, length x width) of the sample (top, bottom, left, right, and center), and the cytocapsular tube density (CCT/mm^2) was calculated and determined for each area. The average CCT density across the 5 sites was treated as the specimen's overall CCT density and round up to digits. The quantitation of a humoral vessel density employs the similar method as CCT quantitation (in **Fig. S12B**).

Supplementary figures and legends:

Fig. S1

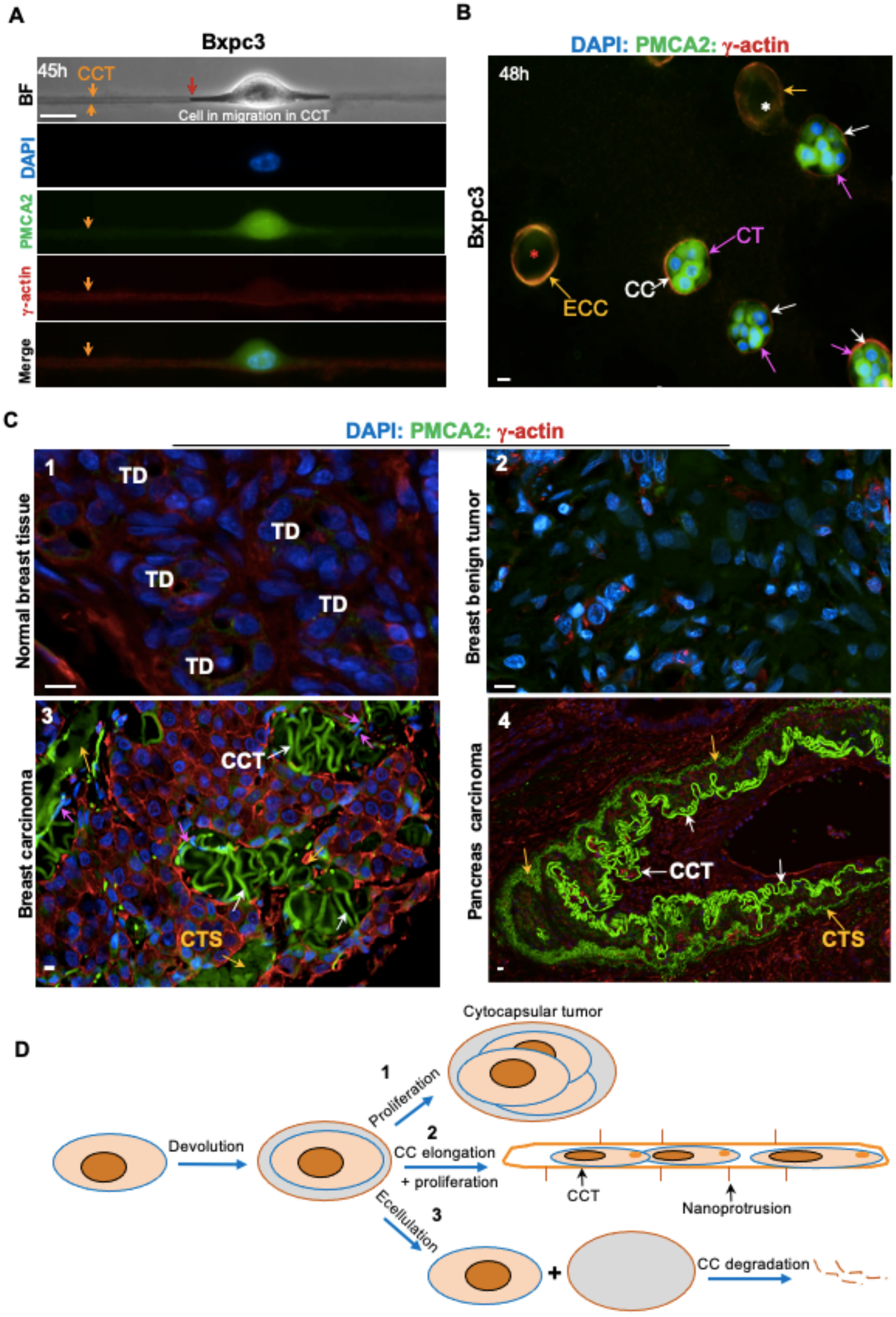

A
Bxpc3
45h
CCT
Cell in migration in CCT
BF
DAPI
PMCA2
γ-actin
Merge
B
DAPI: PMCA2: γ-actin
48h
Bxpc3
CT
CC
ECC
C
DAPI: PMCA2: γ-actin
Normal breast tissue
TD
Breast benign tumor
Breast carcinoma
CCT
CTS
Pancreas carcinoma
D
Cytocapsular tumor
Proliferation
Devolution
CC elongation
+ proliferation
CCT
Nanoprotrusion
Ecellulation
CC degradation

Fig. S1. Detection and lifecycle of cytocapsular oncocells. (*A*) Representative bright filed (BF) and immunohistochemistry (IHC) microscope images of cytocapsular tube (CCT) in CC/CCT culture kit matrix *in vitro*. There is a single Bxpc3 cancer cell in migration in the CCT. CCT membrane edges (orange arrows) are shown. (*B*) Representative immunohistochemistry microscope image of cytocapsular tumorsphere (CT, purple arrows) in CC/CCT culture kit matrix *in vitro*. Cytocapsula (CC, white arrow), ecellulated CC (ECC, orange arrows) and ECC with open holes (white asterisk) are shown. (*C*) Representative immunohistochemistry microscope image of human normal **1**, benign **2** and cancer tissues **3**, **4** with CC/CCT detection by anti-PMCA2 antibodies and anti-γ-actin antibodies. Terminal duct (TD), cytocapsular tube (CCT, white arrows), CCT strand (CTS, orange arrows) are shown. (*D*) Schematic diagram of lifecycle of cytocapsular oncocell: transformed (cancerous) cells experience CC generation and perform cytocapsulasome-driven cytocapsula formation. Cytocapsulas wrap oncocells inside and form cytocapsular oncocells. **1** cytocapsular oncocells proliferate and grow into cytocapsular tumors. **2** Cytocapsular oncocells proliferate and cytocapsulas elongate and develop into CCTs. **3** Ecellulation of cytocapsular oncocells produces acellular cytocapsulas and acytocapsular oncocells. Acellular cytocapsulas and CCTs perform autodegradation and decomposition and disappearance. Scale bar, 10μm.

Fig. S2

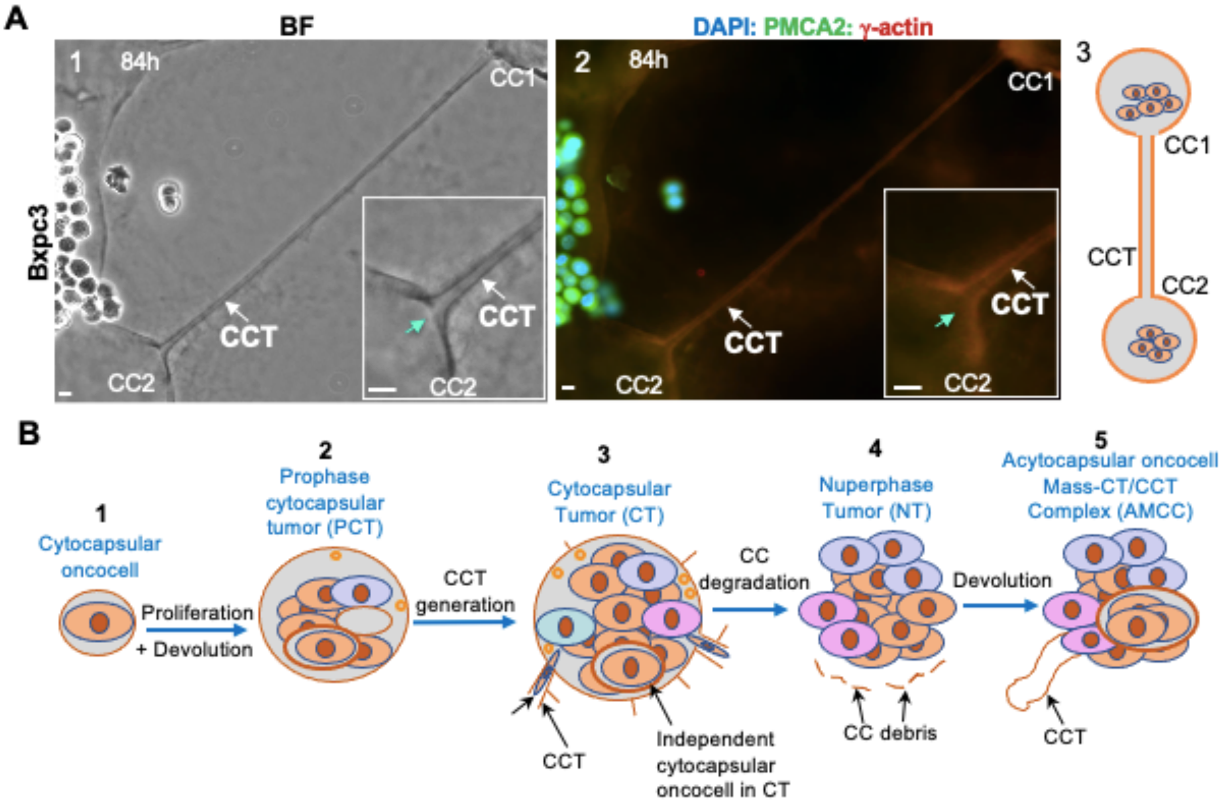

Fig. S2. (*A*) Representative bright field (BF, panel **1**) and fluorescence microscope (panel **2**) images of the same imaged area show that a long, straight, and stretched CCT (white arrows) links two CCs of two cytocapsular tumorspheres (CTs) with open ends in both connection sites. Cytocapsular tube (CCT, white arrows), cytocapsulas (CC1 and CC2) of the two CTs, and open end (cyan arrows) of CCT are shown. Panel **3** show a schematic diagram of two cytocapsulas interconnect by a CCT with two ends open. (*B*) Schematic diagram of lifecycle of cytocapsular tumor: **1** Generation of cytocapsular oncocells; **2** cytocapsular oncocells proliferate and grow into prophase cytocapsular tumor (PCT) without CCTs. Incytocapsular oncocells may experience CC generation and engender independent CCs in the PCT cytocapsular lumen. **3** PCT develops into CT with CCTs. CCTs provide physical membrane-enclosed freeways for incytocapsular oncocell metastasis. **4** CCs of cytocapsular tumors and CCTs degrade, and form acytocapsular oncocell

masses of NT. **5** Some acytocapsular oncocells experience CC generation in the stressful microenvironments in NTs, and generate cytocapsular oncocells, which engender CCTs, and form Acytocapsular oncocell Mass-CT/CCT Complex (AMCC). Acytocapsular oncocells around CCTs invade into CCTs via alloentry and proceed to cancer metastasis. Scale bar, 10μm.

Fig. S3

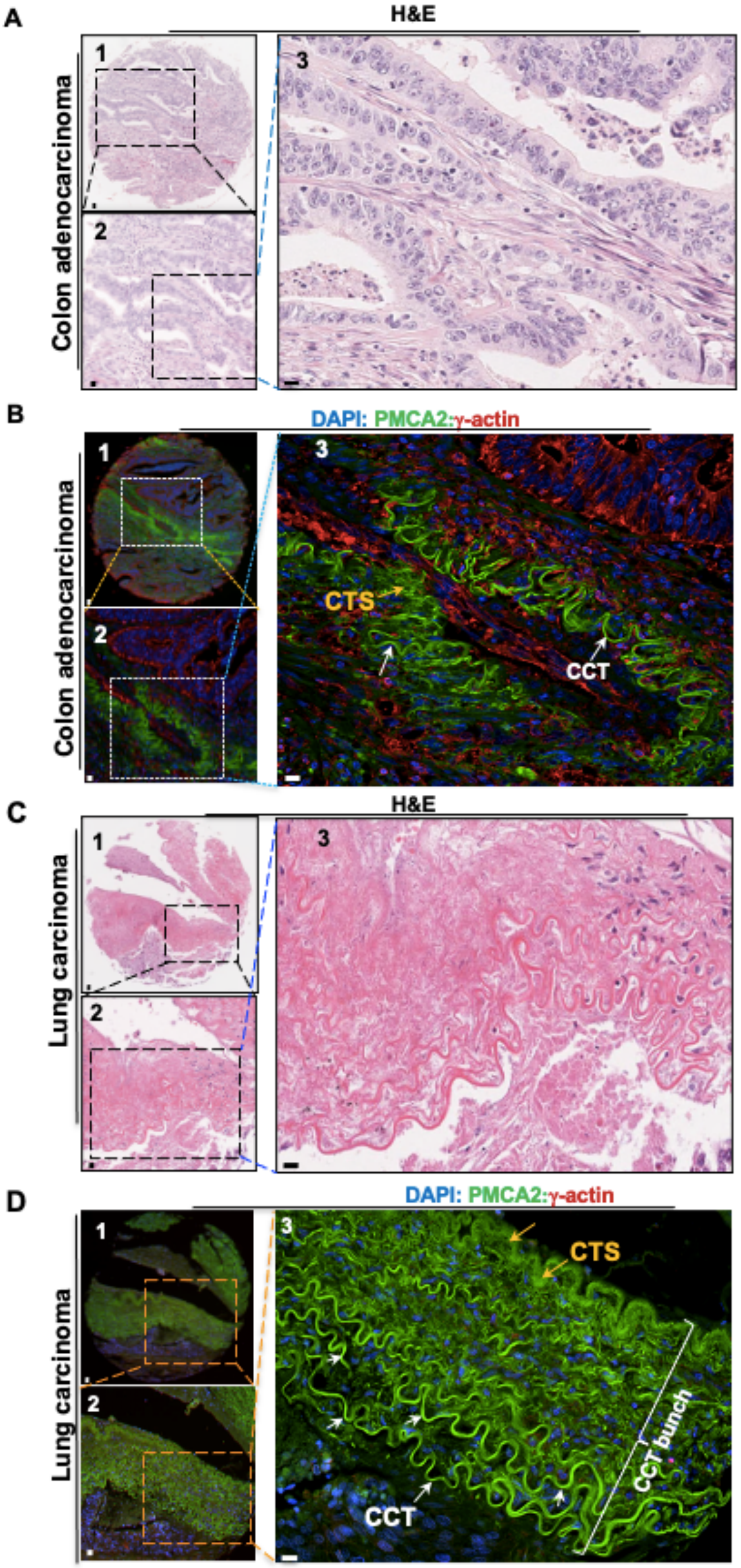
A
H&E
Colon adenocarcinoma
1
2
3
B
DAPI: PMCA2:γ-actin
Colon adenocarcinoma
CTS
CCT
C
H&E
Lung carcinoma
D
DAPI: PMCA2:γ-actin
Lung carcinoma
CTS
CCT
CCT bunch

Fig. S3. Detection of CCTs by IHC fluorescence staining with anti-PMCA2 antibodies. (*A-B*) Representative image of H&E staining (A) and IHC fluorescence staining with anti-PMCA2 antibodies (B) of two continuously sectioned and neighboring colon carcinoma tissue specimens. Panel **1** is the microscope image of a whole colon carcinoma core. The framed area in panel **1** is enlarged and shown in panel **2**. The framed area in panel **2** is enlarged and shown in panel **3**. (*C-D*) Representative image of H&E staining (C) and IHC fluorescence staining with anti-PMCA2 antibodies (D) of two continuously sectioned and neighboring lung carcinoma tissue specimens. CCTs in colon and lung carcinoma tissues are invisible in H&E technologies (panels A and C), but are clearly visible in images with IHC fluorescence staining with anti-PMCA2 antibodies (panels B and D). Cytocapsular tube (CCT, white arrows), and CCT strand (CTS, orange arrows). Scale bar, 10μm.

Fig. S4

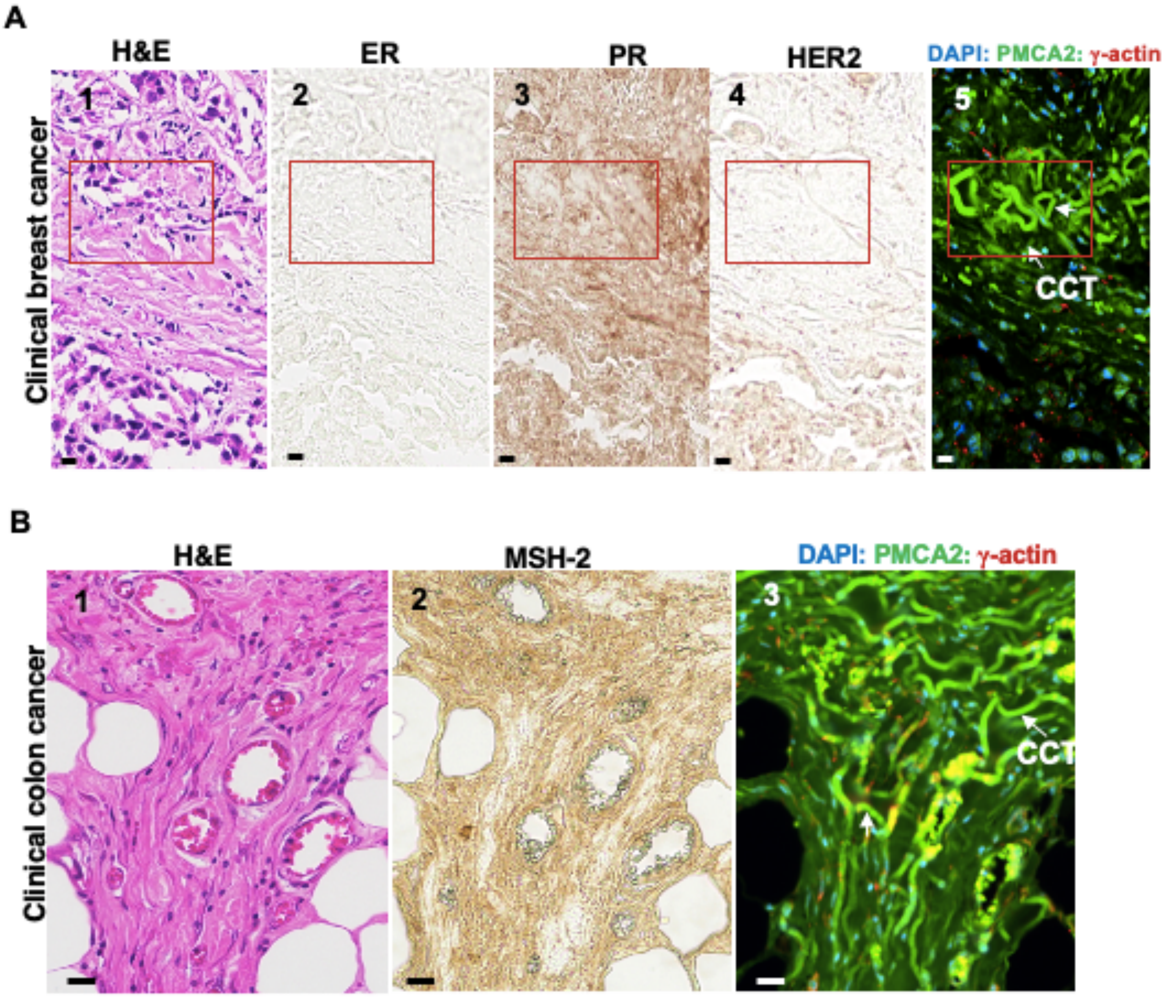

Fig. S4. Detection of CCTs, CCT formation and cytocapsular tumor lifecycle. (*A*) Representative image of H&E staining **1**, IHC staining with antibodies recognizing breast cancer molecular markers ER **2**, PR **3**, and HER **4**, and IHC staining with anti-PMCA2 antibodies **5** of 5 pieces of continuously sectioned breast cancer tissue specimens. The red framed areas in the same site of each tissue specimen show that CCTs (white arrows) presented in panel **5** are invisible in the panels 1-4. (*B*) Representative image of H&E staining **1**, IHC staining with antibodies recognizing colon cancer molecular markers MSH-2 **2**, and IHC staining with anti-PMCA2 antibodies **3** of 3 pieces of continuously sectioned colon cancer tissue specimens. The large quantities of CCTs (white arrows) presented in panel **3** are undetectable in panels **1** and **2**.

Fig. S5

Characterization of acytocapsular oncocell, cytocapsular oncocell, cytocapsular tumor, and cytocapsular tumor network system in 293 types/subtypes of cancers in human organs and tissues.

	Cancer types by organs/ tissues	*Cancer subtypes	Tissue specimen number	Patient number	Male	Female	Age	Cancer stages	With acytocap-sular oncocell	With cytocapsular oncocell in each checked subtype	With CCTs in each checked subtype	With cytocapsular tumors in each checked subtype	With CCT network in each checked subtype	With CTNS in each checked subtype
1	Adrenal Gland	3	23	22	10	12	26-71	I-IV	+	+	+	+	+	+
2	Bladder	7	46	46	30	16	36-74	I-IV	+	+	+	+	+	+
3	Blood (in blood vessel)	3	14	14	8	6	21-45	I-IV	+					
4	Blood (in bone marrow)	2	35	35	18	17	35-70	I-IV	+	+	+		+	
5	Bone	12	212	210	115	95	21-78	I-IV	+	+	+	+	+	+
6	Bone marrow	5	20	20	10	10	25-64	I-IV	+	+	+		+	
7	Brain	8	124	122	86	36	24-67	I-IV	+	+	+	+	+	+
8	Breast	49	3960	3890		3890	18-86	In situ	+	+	+	+	+	+
								I	+	+	+	+	+	+
								II	+	+	+	+	+	+
								III	+	+	+	+	+	+
								IV	+	+	+	+	+	+
9	Cervix	7	53	51		51	37-55	I-IV	+	+	+	+	+	+
10	Colon	6	1586	1586	823	763	16-90	I-IV	+	+	+	+	+	+
11	Esophagus	7	63	62	41	21	24-65	I-IV	+	+	+	+	+	+
12	Fibrous	7	25	25	12	13	35-67	I-IV	+	+	+	+	+	+
13	Gallbladder	4	26	26	20	6	37-62	I-IV	+	+	+	+	+	+
14	Head/neck	5	12	12	4	8	41-66	I-IV	+	+	+	+	+	+
15	Intestine	3	15	15	10	5	36-68	I-IV	+	+	+	+	+	+
16	Kidney	11	123	120	81	39	33-65	I-IV	+	+	+	+	+	+
17	Liver	8	362	358	231	127	36-76	I-IV	+	+	+	+	+	+
18	Lung	30	683	676	437	239	27-78	I-IV	+	+	+	+	+	+
19	Lymph	13	56	56	31	25	21-73	I-IV	+	+	+	+	+	
20	Oral cavity	6	17	17	10	7	28-69	I-IV	+	+	+	+	+	+
21	Ovary	6	83	83		83	32-61	I-IV	+	+	+	+	+	+
22	Pancreas	16	222	218	162	56	28-74	I-IV	+	+	+	+	+	+
23	Penis	3	3	3	3		45-62	I-IV	+	+	+	+	+	+
24	Prostate	7	1606	1528	1528		35-77	I-IV	+	+	+	+	+	+
25	Rectum	7	5	5	4	1	32-57	I-IV	+	+	+	+	+	+
26	Skin	10	122	122	35	87	21-79	I-IV	+	+	+	+	+	+
27	Spleen	3	6	6	1	5	36-48	I-IV	+	+	+	+	+	
28	Smooth muscle	3	20	20	10	10	35-55	I-IV	+	+	+	+	+	+
29	(other) Soft tissues	12	120	106	56	50	36-66	I-IV	+	+	+	+	+	+
30	Stomach	10	245	245	123	122	36-72	I-IV	+	+	+	+	+	+
31	Testis	5	24	24	24		41-67	I-IV	+	+	+	+	+	+
32	Thymus	8	5	5	4	1	37-56	I-IV	+	+	+	+	+	
33	Thyroid	3	17	17	10	7	29-61	I-IV	+	+	+	+	+	+
34	Uterus	1	26	26		26	35-56	I-IV	+	+	+	+	+	+
35	Vulva	3	13	13		13	32-55	I-IV	+	+	+	+	+	+
	**Total**	**293**	**9972**	**9784**	**3937**	**5847**								

(* Checked cancer subtypes are listed in Table S2.)

Fig. S5. Characterization of oncocell, acytocapsular oncocell, cytocapsular oncocell, cytocapsular tumor, and cytocapsular tumor network system in 293 types/subtypes of cancers in human organs and tissues.

Fig. S6

A

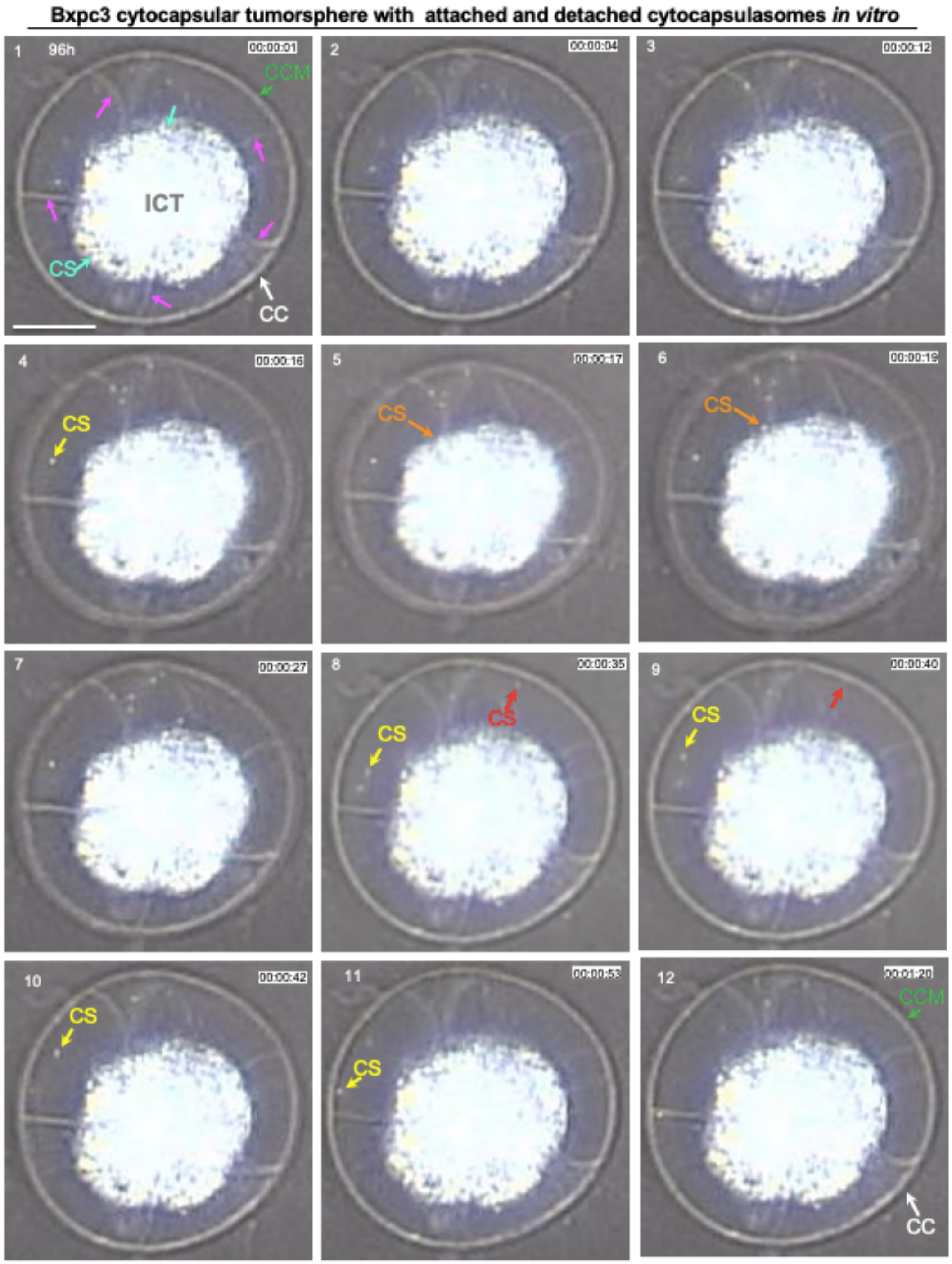

Bxpc3 cytocapsular tumorsphere with attached and detached cytocapsulasomes *in vitro*
1
96h
00:00:01
CCM
ICT
CS
CC
2
00:00:04
3
00:00:12
4
00:00:16
CS
5
00:00:17
CS
6
00:00:19
CS
7
00:00:27
8
00:00:35
CS
CS
9
00:00:40
CS
10
00:00:42
CS
11
00:00:53
CS
12
00:01:20
CCM
CC

B

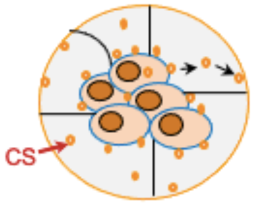

CS

Fig. S6. Cytocapsulasomes drives cytocapsula growth. (*A*) Real-time analysis of cytocapsula growth driven by cytocapsulasomes with a bright field phase contrast microscope. The images are taken from **Movie S1**. Cytocapsulasome activity procedures: **1** cytocapsulasomes (CS) are generated and released by oncocells, and attach to the outside cytoplasm membrane (cyan arrows); **2** CSs spontaneously are detached from oncocell surface (panels **5** and **6**, orange arrows); **3** detached CSs randomly move in the cytocapsular lumen fluids (yellow arrows in panel **4, 8, 9, 10, 11**); **4** CSs reach the inner side of cytocapsular membrane of CT, and contact and integrate into cytocapsular (CC) membrane of CT, and increase CC membrane size in area (red arrows, panels **8** and **9**). In cytocapsular tumors with cytocapsula tightly wrapped oncocell mass surfaces, the contact and integration of cytocapsulasomes into CC membranes will be faster and more efficient without random and long movement journey in the cytocapsular lumen fluids. Incytocapsular tumorsphere (ICT), cytocapsula (CC, white arrows), cytocapsular membrane (CCM, green arrow), cytocapsular spike (purple arrows), and cytocapsulasome (CS; yellow arrow, CS in random movement in cytocapsular lumen fluid; orange arrow, CS detaching from oncocell surface; and red arrows, CS reach and fuse into CC membrane; cyan arrow: CS attached on oncocell surface) are shown. (*B*) Schematic diagram of a cytocapsula tumorsphere with many cytocapsulasomes and spike-like structures in lumen, and cytocapsulasomes are detached from the oncocell surface and move to and fuse into the enlarged CC supporting CC growth. Scale bar, 10μm.

Fig. S7

A

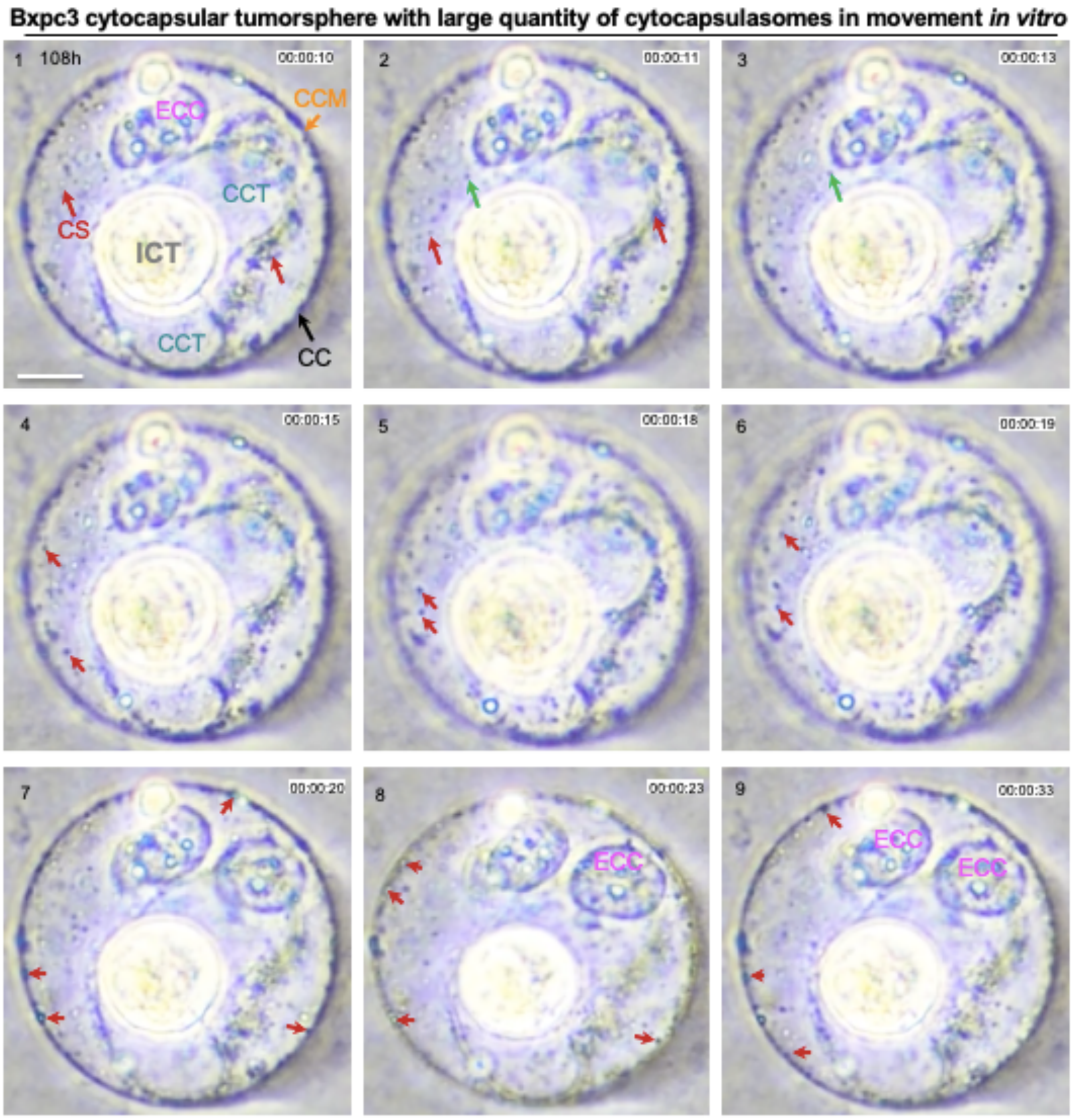
Bxpc3 cytocapsular tumorsphere with large quantity of cytocapsulasomes in movement *in vitro*
1 108h
00:00:10
ECC
CCM
CCT
CS
ICT
CCT
CC
2
00:00:11
3
00:00:13
4
00:00:15
5
00:00:18
6
00:00:19
7
00:00:20
8
00:00:23
ECC
9
00:00:33
ECC
ECC

B

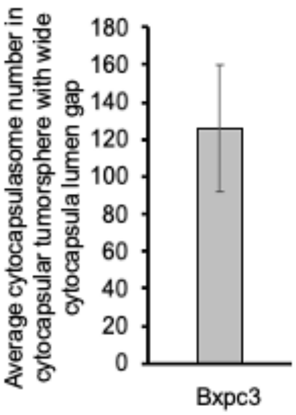
Average cytocapsulasome number in cytocapsular tumorsphere with wide cytocapsula lumen gap
180
160
140
120
100
80
60
40
20
0
Bxpc3

C

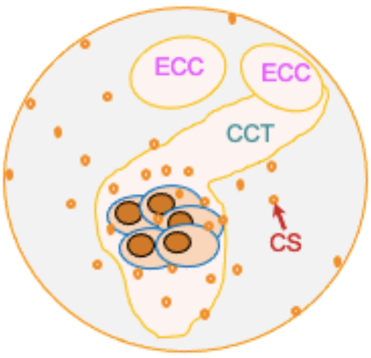
ECC
ECC
CCT
CS

Fig. S7. Large quantities of cytocapsulasomes promote cytocapsular growth. (*A*) Real-time analysis of cytocapsula growth driven by cytocapsulasomes with a bright field phase contrast microscope. The images are taken from **Movie S2**. Incytocapsular tumorsphere (ICT), cytocapsula (CC, black arrows), cytocapsula membrane (CCM, orange arrow), cytocapsulasome (CS, red and green arrows), ecellulated cytocapsula (ECC), and cytocapsular tube (CCT, in the enlarged CC lumen) are shown. (*B*) Quantitation of cytocapsulasomes in cytocapsular tumorspheres. (*C*) Schematic diagram of a tumorsphere generates a large CC with many cytocapsulasomes in the lumen. The incytocapsular oncocell mass engender a secondary, big and "L"-shaped CCT in the big CC lumen. There are two middle acellular CCs in the big CC lumen. Scale bar, 10μm.

Fig. S8

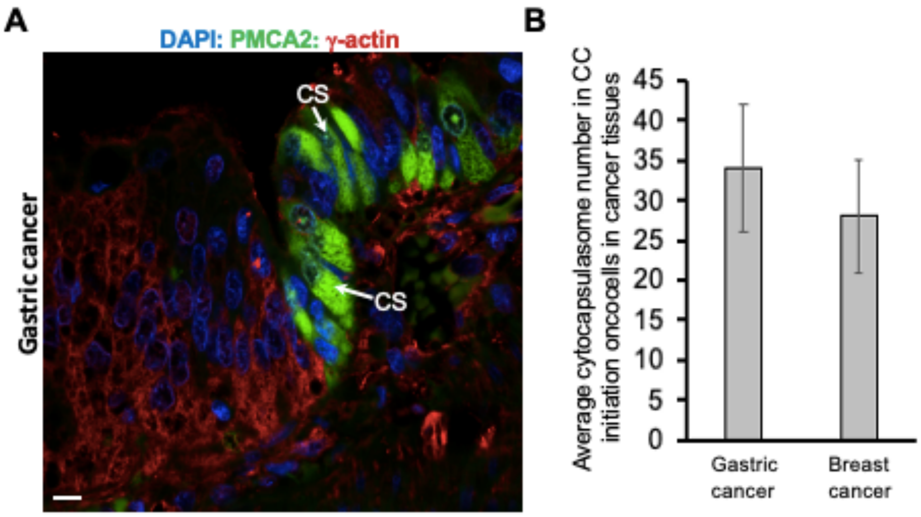

C

Comparative differences and similarities between cytocapsulasome and other 4 extracellular vesicles.

Feature	Cytocapsulasome	Exosome	Apoptotic body	Microvesicle	Large oncosome
Topology	Sphere	Sphere	Sphere or irregular morphology	Sphere or irregular morphology	Sphere or irregular morphology
Membrane enclosed vesicles	+	+	+	+	+
If merge to form cytocapsula	+	-	-	-	-
If merge to from cytocapsular tube	+	-	-	-	-
Homogeneous or heterogeneous in size	Heterogeneous	Homogeneous	Heterogeneous	Heterogeneous	Heterogeneous
Size in diameter/width	40-500nm	30-100 nm	1–5 μm	100-1000 nm	1-10 μm
Localization	In CC/CCT lumen	Outside cell		Outside cell	Outside cell
Cell type that generates vesicle	Cytocapsular oncocell	Cell	Cell	Cell	Cell
Marker proteins	PMCA2	Membrane impermeable (PI negative) CD63, TSG101, Alix	Membrane permeable (PI positive) Annexin V		EGFRvIII
Destinations	Inner side of CC and CCT membranes	Other cells	Intercellular spaces	Other cells	Other cells
Biological functions	CC and CCT building blocks，ECM degradation, and other functions	Intercellular communications	Cellular apoptosis	Intercellular communications	Transferring tumor-promoting material

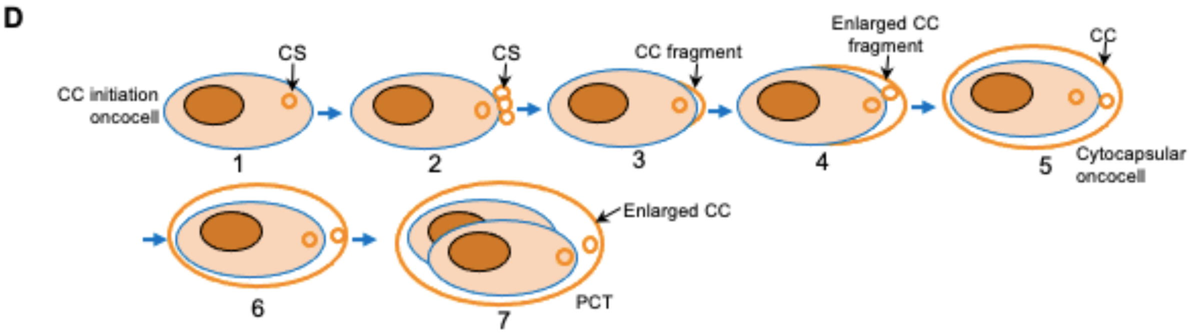

Fig. S8. Characterization of cytocapsulasome and its lifecycle. (*A*) Representative image of cytocapsulasomes (CSs, white arrows) in CC initiation gastric cancer cells during the generation of cytocapsular oncocells *in vivo*. There are many CSs in the cytoplasm. (*B*) Quantitation of cytocapsulasomes in gastric and breast cancer cells with cytocapsulasomes *in vivo*. (*C*) Comparative differences and similarities between cytocapsulasome and other 4 extracellular vesicles. (*D*) Schematic diagram of cytocapsulasome lifecycle: **1** Generation of CS in the cytoplasm of CC initiation oncocells, **2** Release of CS onto the outside of the cytoplasm membrane, and attach to the cell membrane surface, **3** Multiple CSs on the cell membrane contact and integrate into cytocapsular membrane fragments, **4** Cytocapsular membrane fragment grows up with more CS integration, **5** Cytocapsular membranes envelope the whole single cell and generate cytocapsular oncocells, and isolate them from the ECM, **6** CSs reach and integrate into cytocapsular membranes and increase cytocapsular membrane areas, **7** Cytocapsulas grow up and generate enlarged cytocapsulas or elongate and develop into cytocapsular tubes. Scale bar, 10μm.

Fig. S9

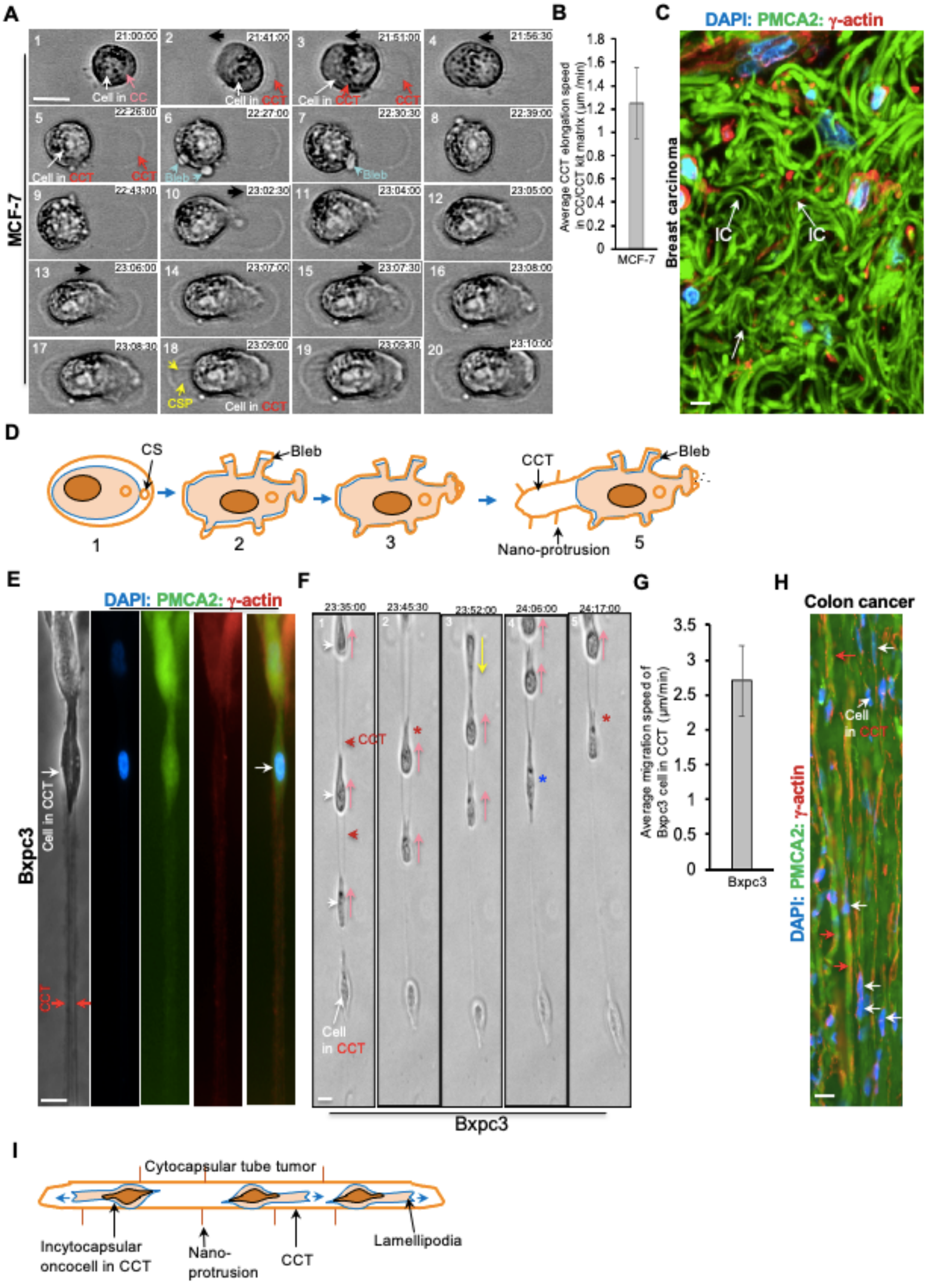
A
MCF-7
Cell in CC
Cell in CCT
CCT
Bleb
CSP
B
Average CCT elongation speed in CC/CCT kit matrix (μm /min)
MCF-7
C
DAPI: PMCA2: γ-actin
Breast carcinoma
IC
D
CS
Bleb
CCT
Nano-protrusion
1
2
3
5
E
DAPI: PMCA2: γ-actin
Bxpc3
Cell in CCT
CCT
F
23:35:00
23:45:30
23:52:00
24:06:00
24:17:00
CCT
Cell in CCT
Bxpc3
G
Average migration speed of Bxpc3 cell in CCT (μm/min)
Bxpc3
H
Colon cancer
DAPI: PMCA2: γ-actin
Cell in CCT
I
Cytocapsular tube tumor
Incytocapsular oncocell in CCT
Nano-protrusion
CCT
Lamellipodia

Fig. S9. Cytocapsular tube generation and elongation, and cell migration in CCTs *in vitro* and *in vivo.* **A** Real-time analysis of cytocapsular tube initiation, generation and elongation. The images are taken from **Movie S3**. Cytocapsular tube (CCT, red arrows), cell in CCT (white arrows), bleb (cyan arrows), cytocapsular spike (CSP, yellow arrows) are shown. **B** Quantitation of cytocapsular tube elongation speed in the CC/CCT culture kit matrix *in vitro*. **C** Representative image of initial cytocapsular tube (IC, white arrows) in breast cancer tissues *in vivo*. **D** Schematic diagram of cytocapsular tube elongation: **1** Incytocapsular oncocell generate and release cytocapsulasomes and drive cytocapsular membrane area increase, **2** cytocapsular oncocells generate many blebs in all directions, sense microenvironments, choose and decide the motility directions, **3** cytocapsulasomes continuously drive cytocapsulas (CC) membrane increase in areas with many blebs in the CC lumen, **4** cytocapsular oncocells move forward, and elongate CCT length, and generate long CCTs. **E** Representative image of cell migration in CCT *in vitro*.

F Real-time analysis of incytocapsular oncocell migration in CCTs. The images are taken from **Movie S4**. Cytocapsular tube (CCT, red arrows), incytocapsular oncocell in migration (white arrows), migration direction (pink arrows), reversed migration direction (yellow arrows), cell with lamellipodia at the leading edge (red asterisk), cell in transition of migration direction in CCT (blue asterisk) are shown. **G** Quantitation of Bxpc3 pancreas cancer cell migration in CCT *in vitro*. **H** Representative images of colon cancer cells in migration in colon CCTs. Colon cancer incytocapsular oncocells in CCTs are in thin, long and spindle-shaped morphologies. Cytocapsular tube (CCT, red arrows), and colon oncocells in migration in CCT (white arrows) are shown. **I** A schematic diagram of cytocapsular tube tumor. Scale bar, 10μm.

Fig. S10

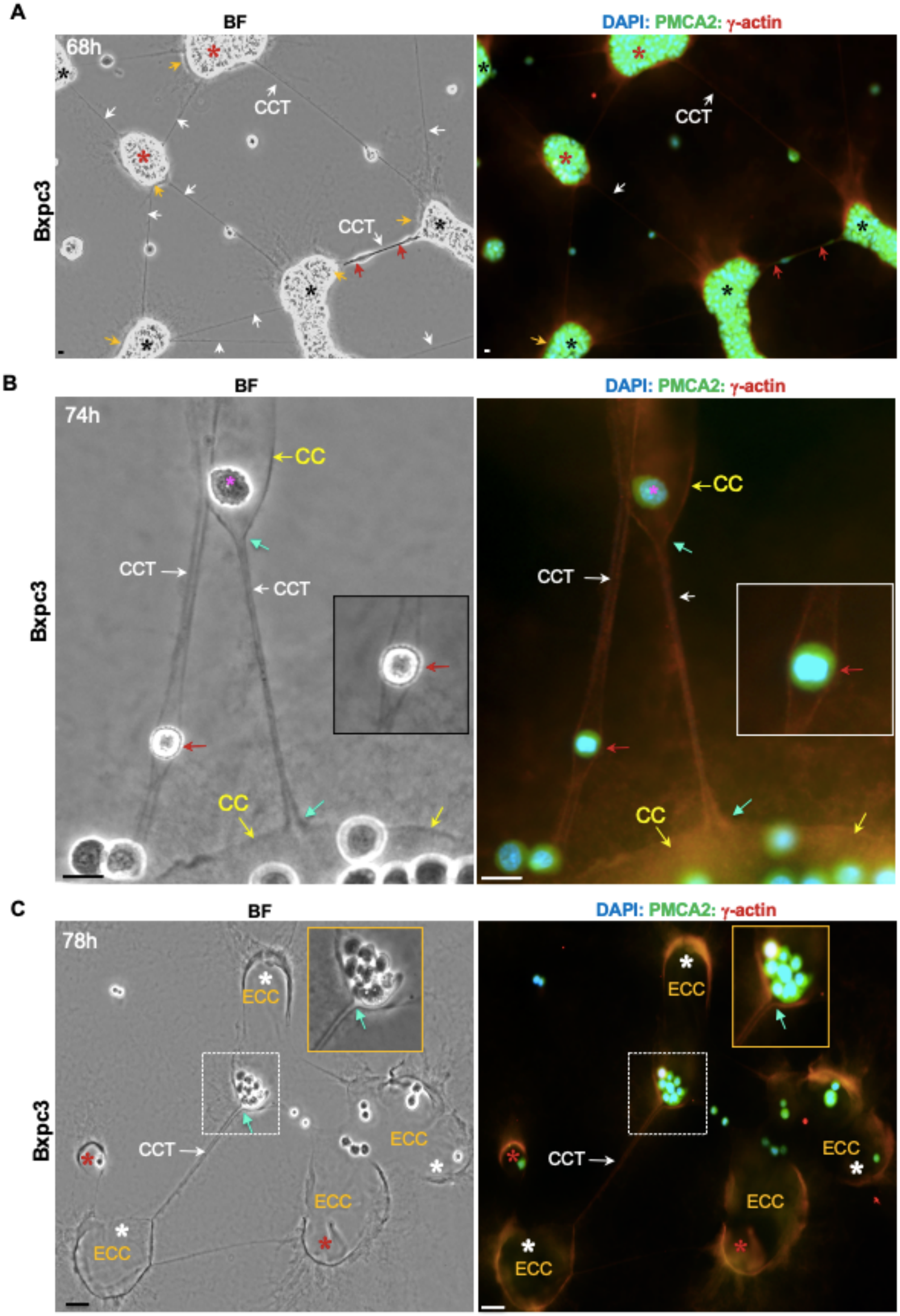

A
BF
DAPI: PMCA2: γ-actin
68h
Bxpc3
CCT
CCT
CCT
B
BF
DAPI: PMCA2: γ-actin
74h
Bxpc3
CC
CCT
CCT
CC
CC
CCT
CC
C
BF
DAPI: PMCA2: γ-actin
78h
Bxpc3
ECC
CCT
ECC
ECC
ECC
ECC
CCT
ECC
ECC
ECC

Fig. S10. Integrated cytocapsular oncocell, cytocapsular tumorspheres, CCT networks, and cytocapsular tumorsphere network systems *in vitro*. **A** Representative image of primary and secondary cytocapsular tumorspheres integrated cytocapsular tumorsphere-network systems (CTNSs) *in vitro*. Firstly, primary cytocapsular tumorspheres (black asterisks) interconnect by CCT networks. Disseminated incytocapsular oncocells gather in the CCT network nodes and grow into secondary cytocapsular tumorspheres (red asterisks). Secondary cytocapsular tumorspheres interconnect primary cytocapsular tumorspheres via CCTs and generate a combined primary and secondary cytocapsular tumorsphere network system in the CC/CCT culture kit (6-well plate). **B** Representative image of cytocapsular tumorsphere interconnection by CCTs with open-ends in both sides of the CCT, and incytocapsular oncocell migration in CCTs and CTNSs. **C** Representative image of membrane-sheltered cytocapsular oncocells, cytocapsular tumorspheres, CCT networks, and integrated CTNSs. After ecellulation, the acellular CCs and CCTs show the interconnected CC and CCT membrane systems of integrated and interconnected primary (white asterisks) and secondary (red asterisks) CT network systems (CTNSs). Cytocapsular tube (CCT, white arrow), cytocapsula (CC, yellow arrows), open-end of both sides of CCTs (cyan arrows) , and incytocapsular oncocell migration in CCTs (red arrows) are shown. Scale bar, 10μm.

Fig. S11

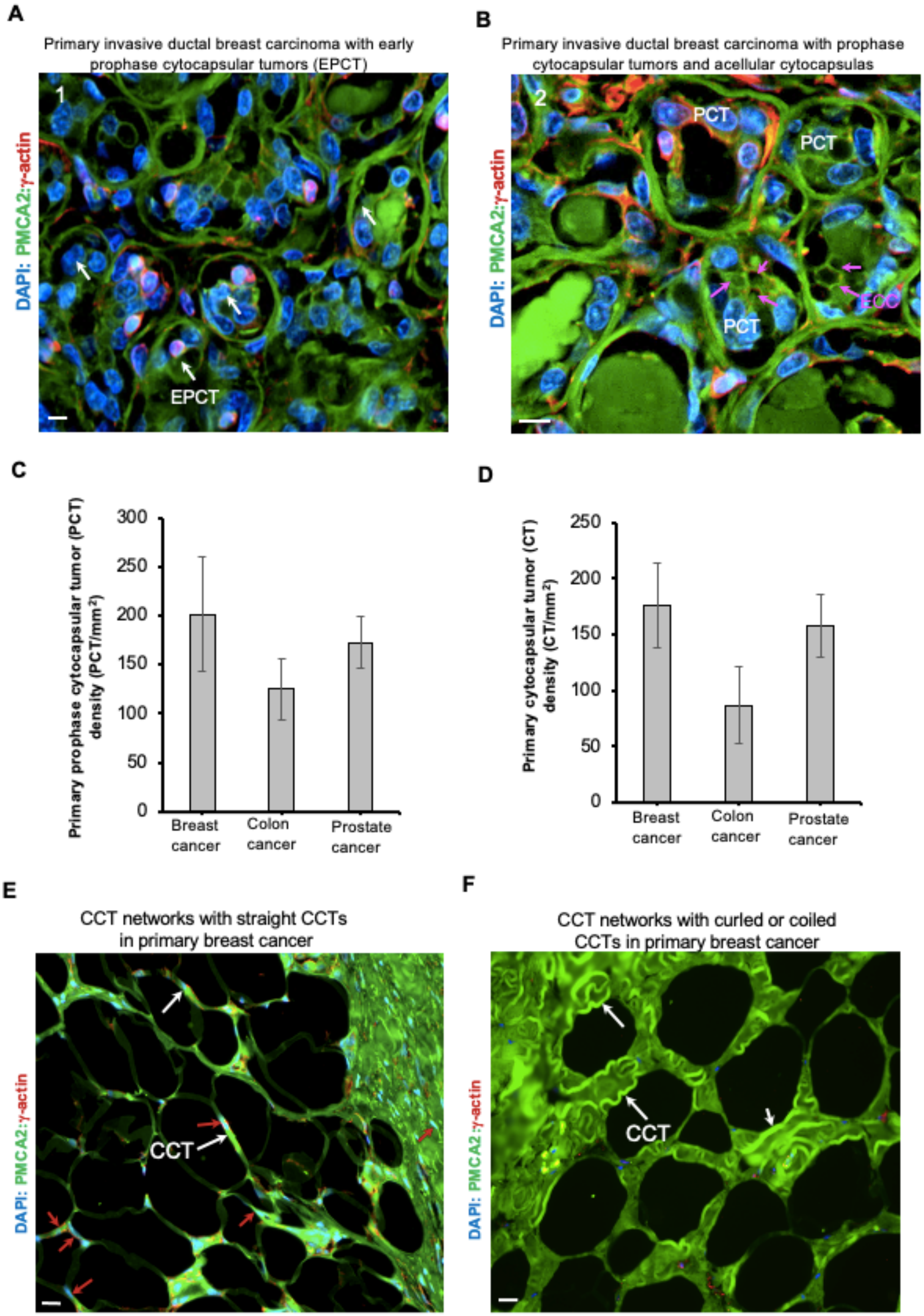
A
Primary invasive ductal breast carcinoma with early prophase cytocapsular tumors (EPCT)
1
DAPI: PMCA2:γ-actin
EPCT
B
Primary invasive ductal breast carcinoma with prophase cytocapsular tumors and acellular cytocapsulas
2
DAPI: PMCA2:γ-actin
PCT
PCT
PCT
ECC
C
Primary prophase cytocapsular tumor (PCT) density (PCT/mm²)
300
250
200
150
100
50
0
Breast cancer
Colon cancer
Prostate cancer
D
Primary cytocapsular tumor (CT) density (CT/mm²)
250
200
150
100
50
0
Breast cancer
Colon cancer
Prostate cancer
E
CCT networks with straight CCTs in primary breast cancer
DAPI: PMCA2:γ-actin
CCT
F
CCT networks with curled or coiled CCTs in primary breast cancer
DAPI: PMCA2:γ-actin
CCT

Fig. S11. Prophase CT, CT and CCT networks *in vivo*. **A** Enlarged image area 1 from **Fig. 4A**. Early prophase cytocapsular tumors (EPCTs, size <50μm and incytocapsular oncocell number <20; white arrows) are shown. **B** Dense PCT and ecellulated cytocapsulas (ECC, purple arrows) in the enlarged CC lumen are shown (enlarged panel from **Fig. 4A**). **C** Quantitation of PCT density in breast, colon and prostate cancers. **D** Quantitation of CT density in breast, colon and prostate cancers. **E** CCT networks composed by straight CCTs in primary breast cancer. **F** CCT networks composed by curled and coiled CCTs in primary breast cancer. Scale bar, 10μm.

Fig. S12

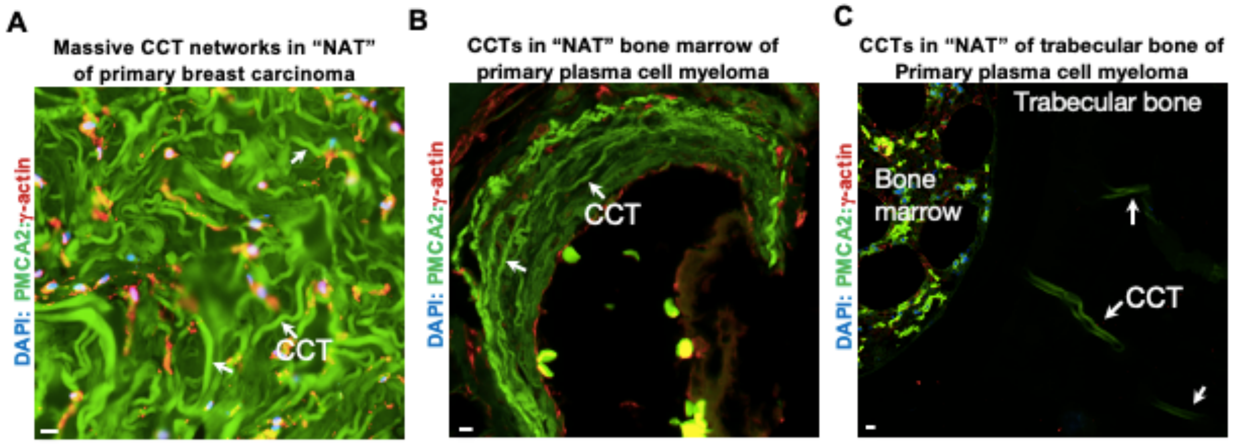

D

Cancer types	Cancer subtypes	NAT tissue specimen number	Number of NAT tissues with CCT	% of paracancer tissue with CCT	CCT density/mm^2
Bladder	2	10	10	100	72-101
Blood cancer in bone marrow	2	20 (in trabecular bone)	20 (in trabecular bone)	100	2-10
Blood cancer in bone marrow	2	10	10	100	35-65
Breast	25	356	356	100	78-114
Esophagus	1	5	5	100	72-103
Colon	7	121	121	100	71-109
Intestine	1	3	3	100	78-107
Kidney	2	6	6	100	75-112
Liver	6	89	89	100	67-103
Lung	5	65	65	100	82-112
Ovary	2	12	12	100	79-114
Pancreas	6	96	96	100	81-107
Stomach	6	78	78	100	79-113
Uterus	1	5	5	100	81-102
Total	68	876	876	100	

Fig. S12. NATs: CCTs are broadly present in native tissue adjacent to tumors (NAT). **A** Representative image of NAT in primary breast carcinoma with large quantities of CCT networks in high density. **B** Representative image of NAT in bone marrow of primary plasma cell myeloma with CCT (white arrows) bunches coexist with immune cells. **C** Representative image of NAT of trabecular bone in primary plasma cell myeloma. CCTs (white arrows) invade into hard tissues of trabecular bone. There is no PMCA2 signal in bone matrices. **D** Quantitation of CCT density in NAT of 14 kinds of tissues. Scale bar, 10μm.

Fig. S13

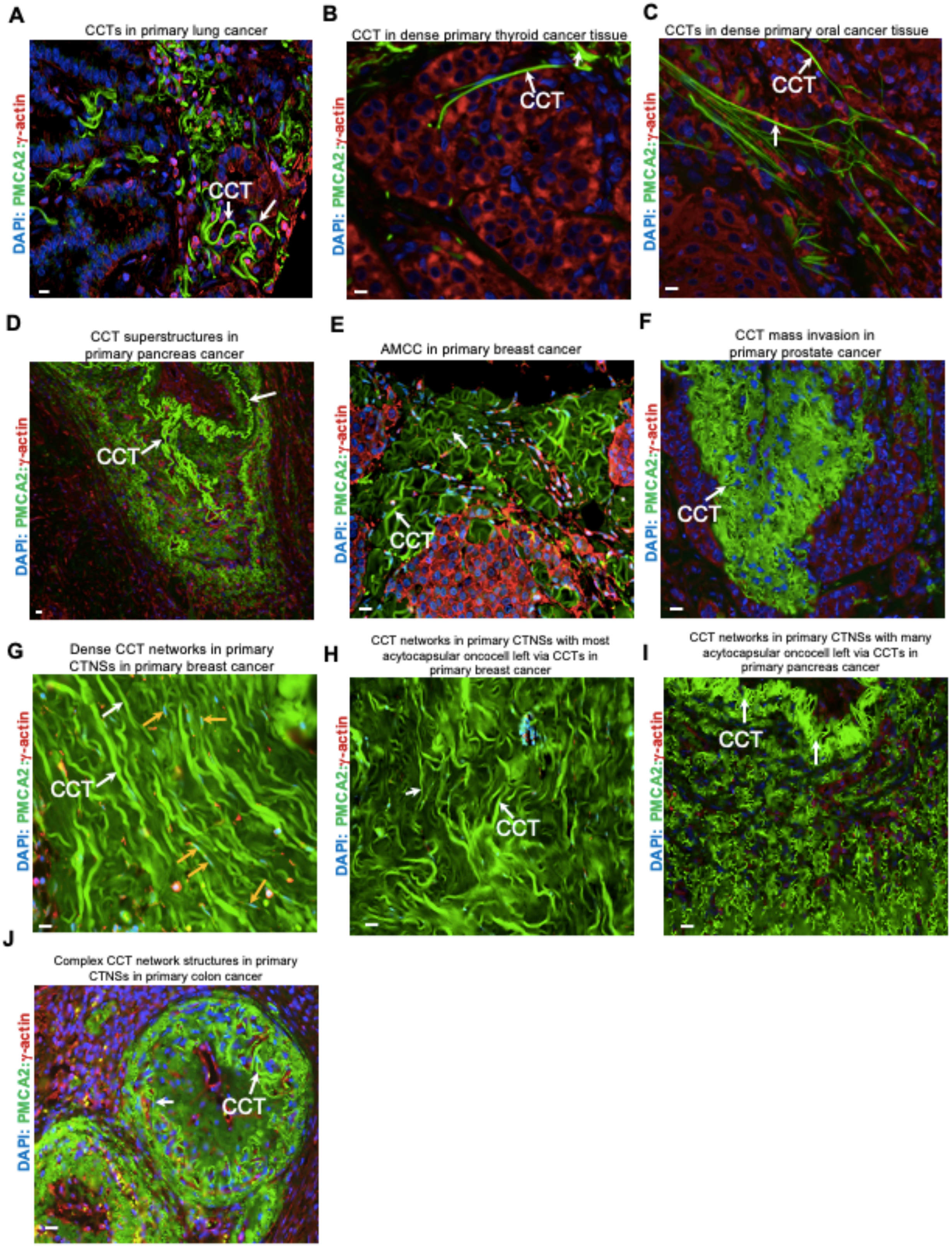
A
CCTs in primary lung cancer
DAPI: PMCA2:γ-actin
CCT
B
CCT in dense primary thyroid cancer tissue
DAPI: PMCA2:γ-actin
CCT
C
CCTs in dense primary oral cancer tissue
DAPI: PMCA2:γ-actin
CCT
D
CCT superstructures in primary pancreas cancer
DAPI: PMCA2:γ-actin
CCT
E
AMCC in primary breast cancer
DAPI: PMCA2:γ-actin
CCT
F
CCT mass invasion in primary prostate cancer
DAPI: PMCA2:γ-actin
CCT
G
Dense CCT networks in primary CTNSs in primary breast cancer
DAPI: PMCA2:γ-actin
CCT
H
CCT networks in primary CTNSs with most acytocapsular oncocell left via CCTs in primary breast cancer
DAPI: PMCA2:γ-actin
CCT
I
CCT networks in primary CTNSs with many acytocapsular oncocell left via CCTs in primary pancreas cancer
DAPI: PMCA2:γ-actin
CCT
J
Complex CCT network structures in primary CTNSs in primary colon cancer
DAPI: PMCA2:γ-actin
CCT

Fig. S13. Cytocapsular oncocell metastasis in primary CTNSs in primary cancer niche. **A** Representative image of CCTs in loose soft tissue in lung cancer. **B-C** CCTs in compact soft tissues in thyroid **B** and oral **C** cancers. **D** CCT superstructures in pancreas cancer.

E Massive curled CCT networks invade into compact acytocapsular oncocell masses and form AMCC. **F** Dense CCT network masses invade through compact primary prostate cancer tissues in AMCC. **G** Representative image of CCT bunches in primary breast carcinoma CTNSs. **H** Representative images of CCT masses in primary breast carcinoma CTNSs and most acytocapsular oncocells left via CCTs. **I** Representative image of highly curled and coiled CCTs in primary CTNSs in primary pancreas cancer with many acytocapsular oncocells left via CCTs. **J** Representative image of complex CCT superstructures in primary CTNSs. CCT (white arrows) and CCT strand (CTS, orange arrows) are shown in panels A-J. Scale bar, 10μm.

Fig. S14

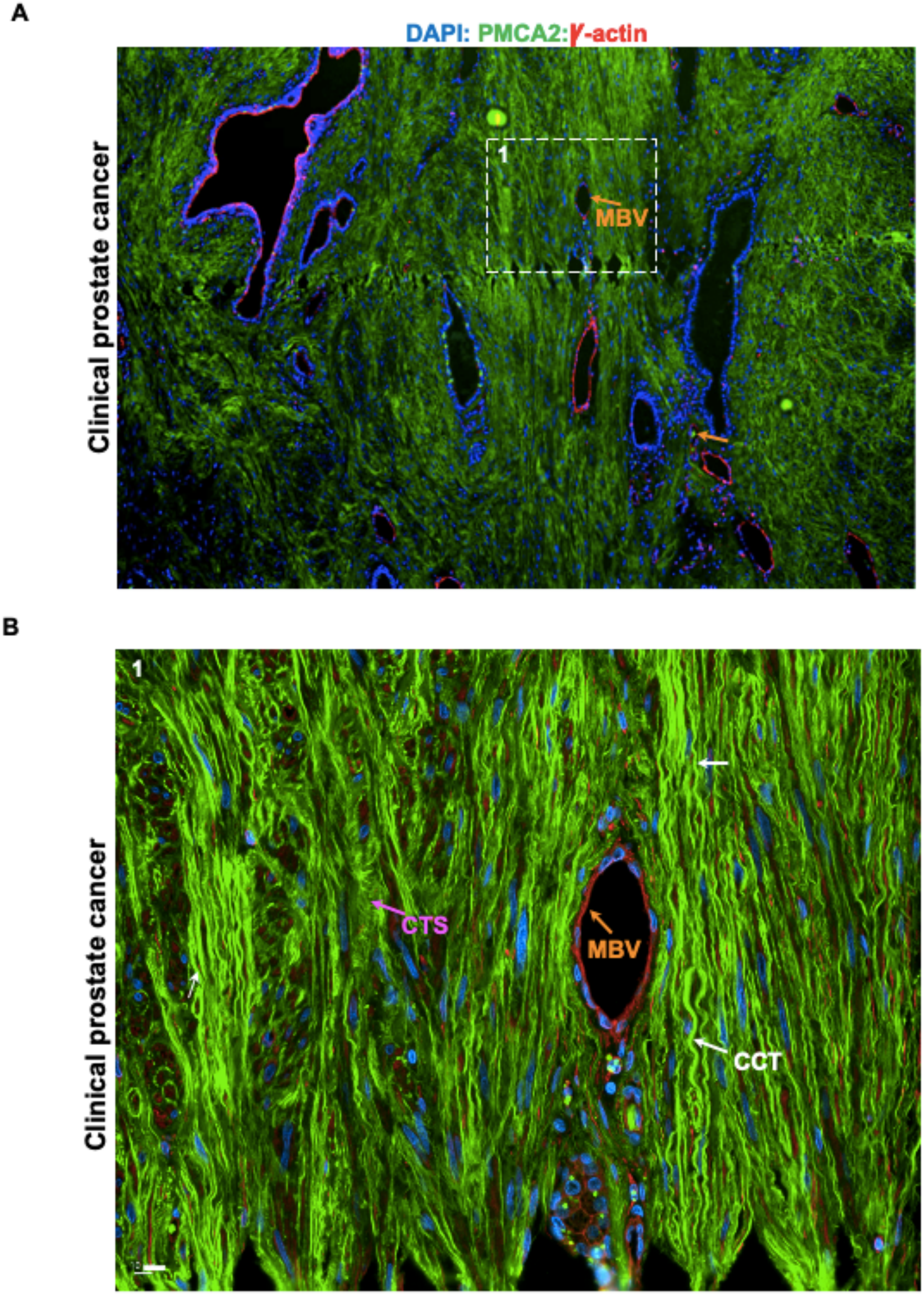
A
DAPI: PMCA2:γ-actin
Clinical prostate cancer
1
MBV
B
1
Clinical prostate cancer
CTS
MBV
CCT

Fig. S14. Large quantities of cytocapsular tube networks beyond humoral vessels dominate cancer metastasis pathways. **A** Representative image of clinical prostate cancer at Stage IIb with large quantities of CCTs and CCT networks occupying most of the spaces beyond humoral vessels (blood vessel, lymph vessels) and prostate grand lumens, and dominate cancer cell metastasis pathways. Most of prostate cancer cells have left away via CCT networks. The white dashed framed area is enlarged in **B**. Micro blood vessels (MBV, orange arrows) are shown. **B** Enlarged image in framed area in **A**. There are large quantities of CCTs (white arrows) and CCT networks beyond the integrated micro blood vessels (MBV). Most of prostate cancer cells have left away via CCT networks. MBVs are intact without damage at this cancer stage. Degraded CCT strands (CTS, red arrow) are shown.

Fig. S15

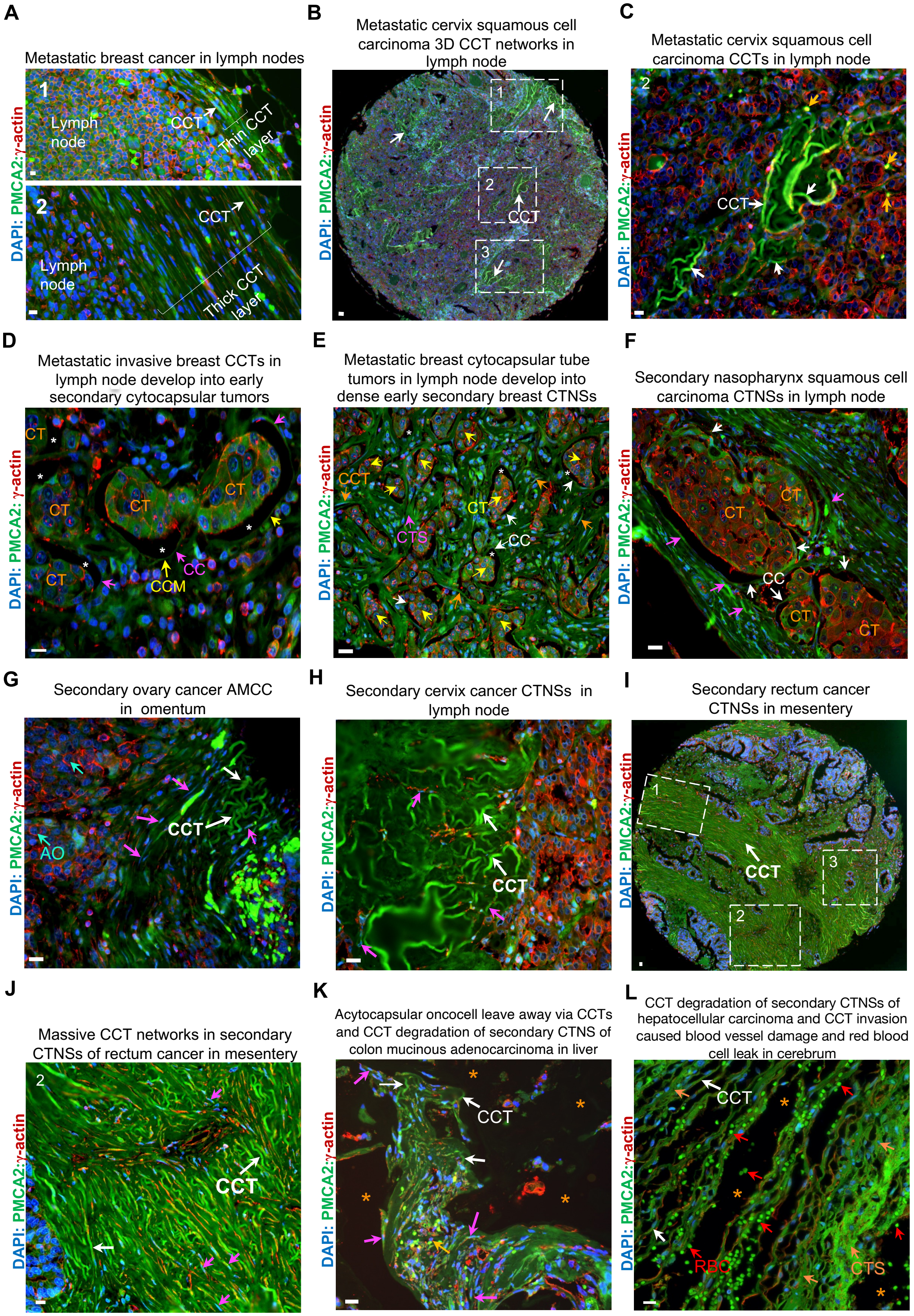

A
Metastatic breast cancer in lymph nodes
1
Lymph node
CCT
Thin CCT layer
2
Lymph node
CCT
Thick CCT layer
DAPI: PMCA2:γ-actin
B
Metastatic cervix squamous cell carcinoma 3D CCT networks in lymph node
1
2
3
CCT
C
Metastatic cervix squamous cell carcinoma CCTs in lymph node
2
CCT
D
Metastatic invasive breast CCTs in lymph node develop into early secondary cytocapsular tumors
CT
CC
CCM
E
Metastatic breast cytocapsular tube tumors in lymph node develop into dense early secondary breast CTNSs
CCT
CTS
CT
CC
F
Secondary nasopharynx squamous cell carcinoma CTNSs in lymph node
CT
CC
G
Secondary ovary cancer AMCC in omentum
CCT
AO
H
Secondary cervix cancer CTNSs in lymph node
CCT
I
Secondary rectum cancer CTNSs in mesentery
1
CCT
3
2
J
Massive CCT networks in secondary CTNSs of rectum cancer in mesentery
2
CCT
K
Acytocapsular oncocell leave away via CCTs and CCT degradation of secondary CTNS of colon mucinous adenocarcinoma in liver
CCT
L
CCT degradation of secondary CTNSs of hepatocellular carcinoma and CCT invasion caused blood vessel damage and red blood cell leak in cerebrum
CCT
RBC
CTS

Fig. S15. Lifecycle of secondary cytocapsular tumor-network systems *in vivo*. (*A*) Representative images of metastatic breast cancer CCT (white arrows) thin (1) and thick (2) bunches wrapping and invading into lymph nodes. (*B*) Metastatic cervix squamous cell carcinoma 3D CCT (white arrows) networks invade into and distributed inside most lymph node areas. White dashed line framed areas 1, 2 and 3 are enlarged in Figs. **S16A, S15C, and S16B**. (*C*) Enlarged white dashed line framed area 2 from Panel (*B*). Metastatic cervix squamous cell carcinoma CCTs invade into lymph node, develop into CCT masses and occupy spaces in lymph nodes. Cross-sectioned CCTs (orange arrows) are shown. (*D*) Metastatic breast CCTs invaded into lymph nodes grow into multiple cytocapsular tumors (CTs). Enlarged cytocapsula (CC, purple arrows), CC membrane (CCM, yellow arrows), and CT lumens (white asterisks) are shown. (*E*) Representative images of secondary breast CTNSs with high CT density in lymph node. Breast CCTs invade into lymph node, incytocapsular oncocells proliferate and grow into many cytocapsular tumors. Large quantities of secondary breast cytocapsular tumors in lymph node interconnect by CCT networks and form dense CTNSs in lymph node. Cytocapsular tumor (CT, yellow arrows), cytocapsula (CC, white arrows), CCT(orange arrows), CCT strand (CTS, purple arrow), and cytocapsular lumens (white asterisks) are shown. (*F*) Degradation of CCs of secondary CTs, CCT degradation, and acytocapsular ovary oncocell uncontrolled proliferation led to enlarged acytocapsular ovary oncocell masses in secondary CTNSs in omentum. (*G*) Some acytocapsular ovary oncocells regenerate CCTs, and form AMCC in secondary CTNSs in omentum. CCT (white arrows), acytocapsular oncocell (AO, cyan arrows), and oncocells in migration in CCTs (purple arrows) are shown. (*H*) Representative image of secondary cervix CTNSs in lymph node with many cervix CCTs in lymph node. CCT (white arrows) and oncocells in migration in CCTs (purple arrows) are shown. (*I*) Representative image of a whole tissue core of secondary rectum CTNSs in mesentery

with high CCT (white arrows) density. White framed areas 1, 2 and 3 are enlarged and shown in **Figs. S16E, S15J and S16F**. (*J*) Enlarged area from (I) to show the high CCT density. CCT (white arrows) and oncocells in migration in CCTs (purple arrows) are shown. (*K*) Secondary colon CTNSs with severe CCT degradation in liver. CCT (white arrows) and oncocells in migration in CCTs (purple arrows) are shown. (*L*) Secondary hepatocellular carcinoma CTNSs with severe CCT degradation, severe blood vessel damage, and severe red blood cell leak, major acytocapsular oncocell metastasis via CCTs (white arrows), and low cell density in the local cerebrum. Red blood cell (RBC, red arrows) randomly distributed outside blood vessels and in tissues, and CCT strand (CTS, orange arrows) are shown. Normal tissue cell apoptosis, acytocapsular oncocell metastasis via CCTs or apoptosis, and CCT degradation caused cavities (black spaces, tissue liquefaction, orange asterisks) in panels K-L are shown. Scale bar, 10μm.

Fig. S16

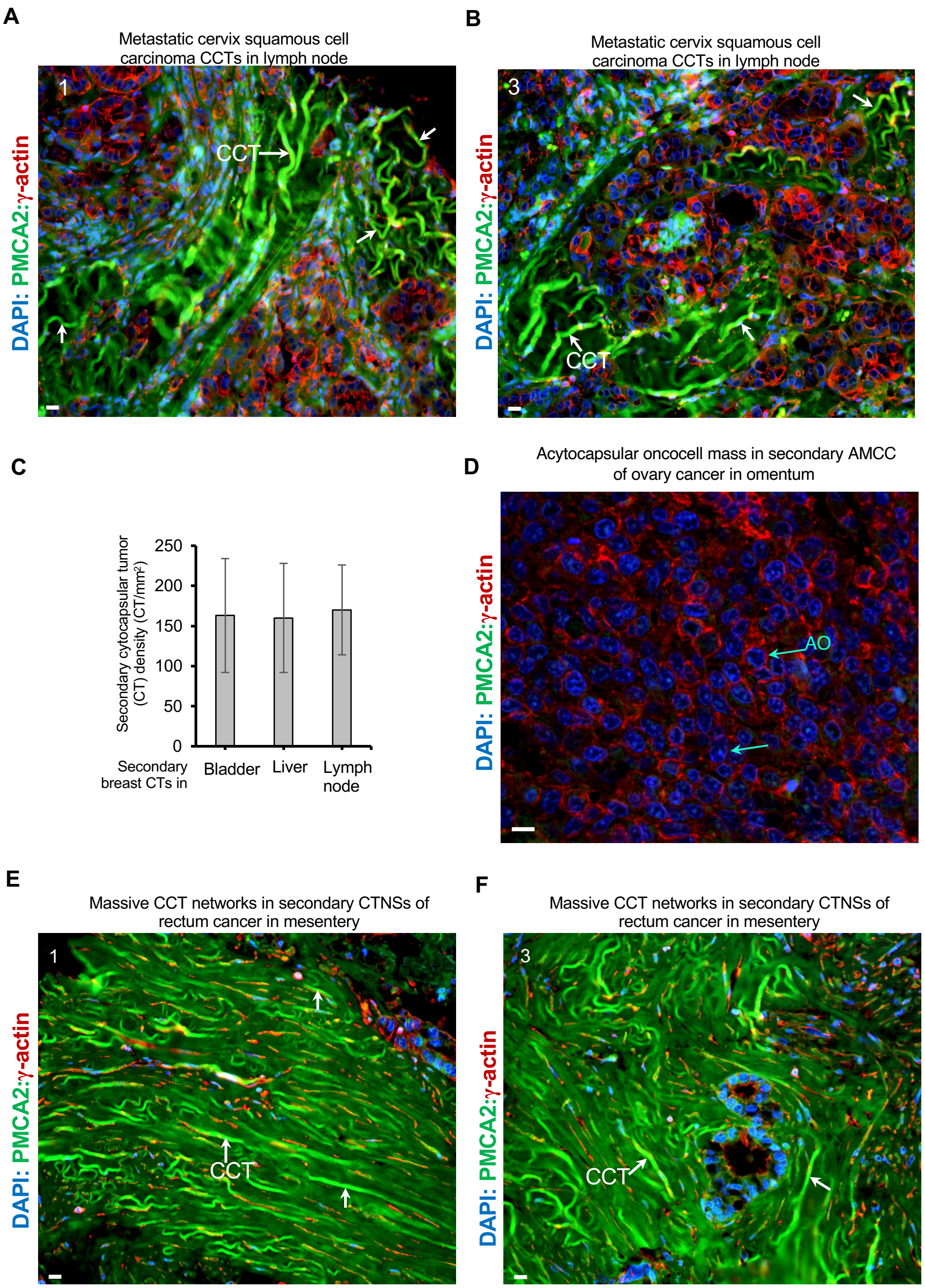
A
Metastatic cervix squamous cell carcinoma CCTs in lymph node
1
CCT
DAPI: PMCA2:γ-actin
B
Metastatic cervix squamous cell carcinoma CCTs in lymph node
3
CCT
DAPI: PMCA2:γ-actin
C
Secondary cytocapsular tumor (CT) density (CT/mm²)
250
200
150
100
50
0
Secondary breast CTs in
Bladder
Liver
Lymph node
D
Acytocapsular oncocell mass in secondary AMCC of ovary cancer in omentum
AO
DAPI: PMCA2:γ-actin
E
Massive CCT networks in secondary CTNSs of rectum cancer in mesentery
1
CCT
DAPI: PMCA2:γ-actin
F
Massive CCT networks in secondary CTNSs of rectum cancer in mesentery
3
CCT
DAPI: PMCA2:γ-actin

Fig. S16. Metastatic CCTs, secondary CT density and secondary AMCC *in vivo*. **A** Enlarged image from white dashed line framed area **1** in **Fig. S15B**. The 3D metastatic cervix squamous cell carcinoma CCTs (white arrows) invade into lymph nodes and form highly curved/coiled CCT bunches and masses and coexist with immune cells in lymph nodes. **B** Enlarged image from white dashed line framed area **3** in **Fig. S15B**. The 3D metastatic cervix squamous cell carcinoma CCTs (white arrows) randomly invade into lymph nodes, coexist with immune cells in lymph nodes, and occupy spaces in lymph nodes. **C** Quantitation analysis of secondary breast CTs in bladder, liver and lymph node. **D** Degradation of CCs of secondary CTs, CCT degradation, and acytocapsular ovary oncocell uncontrolled proliferation led to enlarged acytocapsular ovary oncocell masses in secondary CTNSs in omentum. Acytocapsular oncocells (AO, cyan arrows) masses are shown. **E** Enlarged image from white dashed line framed area **1** in **Fig. S17I.** There are very dense and massive CCT network bunches in secondary CTNSs of rectum cancer in mesentery.

F Enlarged image from white dashed line framed area **3** in **Fig. S17I**. There are very dense CCT network masses in secondary CTNSs of rectum cancer in mesentery.

Fig. S17

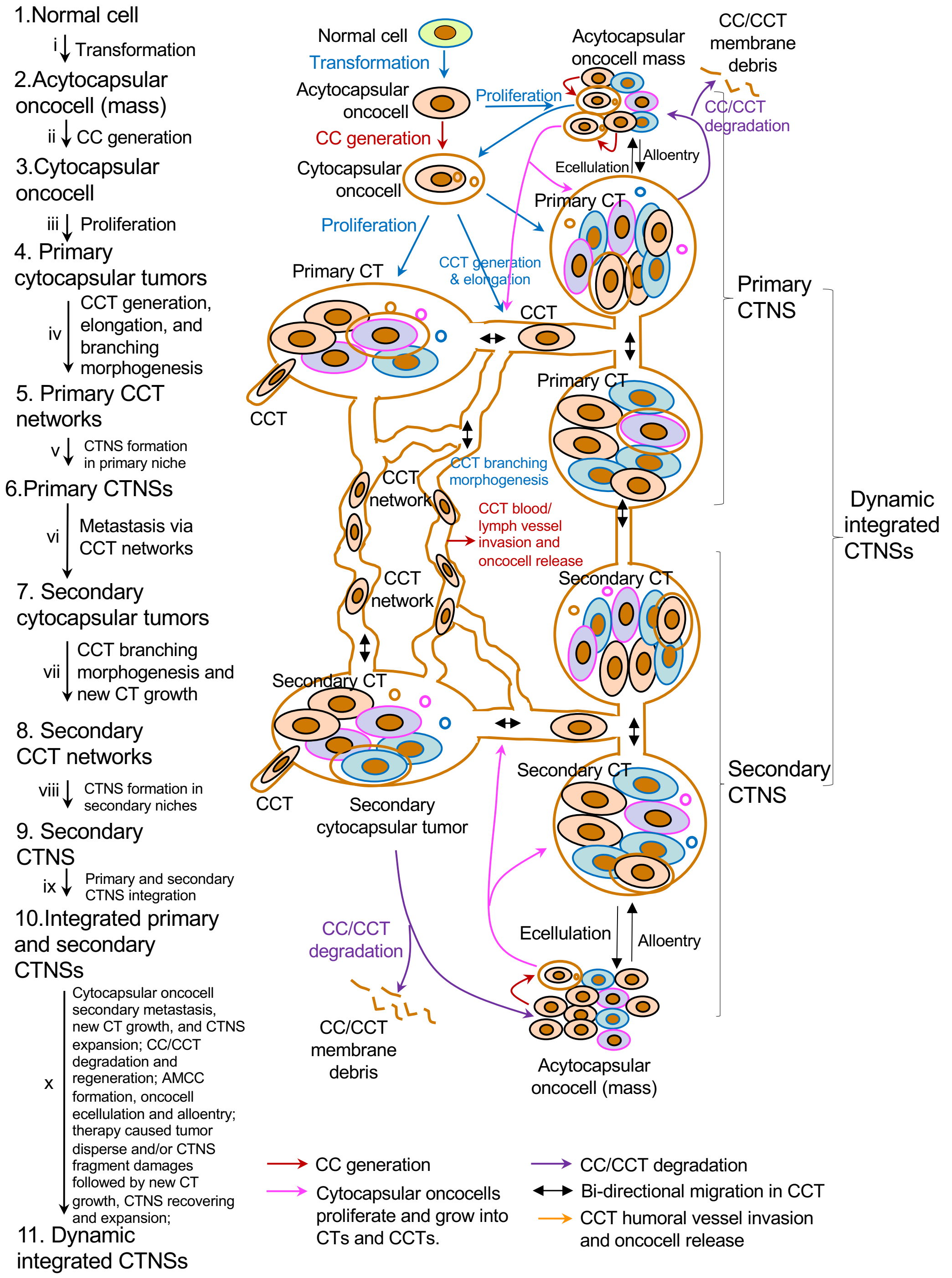
1.Normal cell
i Transformation
2.Acytocapsular oncocell (mass)
ii CC generation
3.Cytocapsular oncocell
iii Proliferation
4. Primary cytocapsular tumors
iv CCT generation, elongation, and branching morphogenesis
5. Primary CCT networks
v CTNS formation in primary niche
6.Primary CTNSs
vi Metastasis via CCT networks
7. Secondary cytocapsular tumors
vii CCT branching morphogenesis and new CT growth
8. Secondary CCT networks
viii CTNS formation in secondary niches
9. Secondary CTNS
ix Primary and secondary CTNS integration
10.Integrated primary and secondary CTNSs
x Cytocapsular oncocell secondary metastasis, new CT growth, and CTNS expansion; CC/CCT degradation and regeneration; AMCC formation, oncocell ecellulation and alloentry; therapy caused tumor disperse and/or CTNS fragment damages followed by new CT growth, CTNS recovering and expansion;
11. Dynamic integrated CTNSs
Normal cell
Transformation
Acytocapsular oncocell
CC generation
Cytocapsular oncocell
Proliferation
Proliferation
Acytocapsular oncocell mass
CC/CCT membrane debris
CC/CCT degradation
Ecellulation
Alloentry
Primary CT
Primary CT
CCT generation & elongation
CCT
CCT
Primary CT
CCT branching morphogenesis
CCT network
CCT blood/ lymph vessel invasion and oncocell release
CCT network
Secondary CT
Secondary CT
Secondary CT
CCT
Secondary cytocapsular tumor
Primary CTNS
Dynamic integrated CTNSs
Secondary CTNS
CC/CCT degradation
Ecellulation
Alloentry
CC/CCT membrane debris
Acytocapsular oncocell (mass)
CC generation
Cytocapsular oncocells proliferate and grow into CTs and CCTs.
CC/CCT degradation
Bi-directional migration in CCT
CCT humoral vessel invasion and oncocell release

Fig. S17. Schematic diagram of an atlas of cytocapsular oncocell evolution lifecycle *in vivo*. **A** simplified atlas of cytocapsular oncocell evolution lifecycle *in vivo* includes 10 steps: **1** Normal cell transformation generates abnormal and acytocapsular oncocell caused by accumulated gene mutation and chemical and physical stimuli from extracellular microenvironments. **2** By unknown molecular mechanisms, some acytocapsular oncocells experience CC generation: generation of cytocapsulasomes and cytocapsulas enclosing the cell and isolating the cell from stressful microenvironments. The additional extracellular protective cytocapsula of cytocapsular oncocell advance to survive under stressful microenvironments. **3** Incytocapsular oncocell proliferate in cytocapsular lumen and generate prophase cytocapsular tumor (PCT) and cytocapsular tumor (CT). **4** CTs in the primary niche generate CCTs, CCT networks, and acytocapsular oncocell mass-CC/CCT complex (AMCC). **5** All CTs in primary niches interconnect by CCT networks and form primary cytocapsular tumor network systems (CTNSs). **6** Primary CCT networks expand and invade into neighboring and far distance tissues and organs. **7** CCT branching morphogenesis, new CCT network formation, and new CT formation and growth in secondary niches. **8** All CTs in the secondary niche interconnect with CCT networks and form secondary CTNSs. AMCC formation in secondary niches. **9** Primary and secondary CTNSs have existed interconnections with CCT metastatic CCT networks, and form integrated primary and secondary CTNSs. **10** A series of activities and responses of CTs, CCTs, CCT networks and CTNSs under various conditions shape the dynamic integrated CTNSs. The expansion and invasion of CCTs, CTs, CTNSs and AMCCs in normal tissues and organs lead to normal cell apoptosis, normal tissue biological function failure and structure damage. Normal cell apoptosis, acytocapsular oncocell apoptosis or leave-away via CCT networks, and CCT degradation result in local cavities filled with intercellular fluids but without tissues (named tissue liquefaction) and tissue/organ biological function failure.

Fig. S18.

Advantages and disadvantages of CC/CCT, CT and CTNS in comparison to other organelles, compartments and cellular activities beyond CC/CCT *in vivo*.

	Characters	CC, CCT, CT, CTNS	Nanotube (Types I and II)	Type II epithelial bridge	Cancer cell activities beyond CC/CCT
1	Uncontrolled cell proliferation inside the organelle/compartment	Yes	No	No	No
2	Cell migration inside the organelle/compartment	Yes	No	No	No
3	Protected cell migration without obstacles of heterogeneous ECM and neighboring cells	Yes	No	No	No
4	Protected cell translocation with immune attack escape	Yes	No	No	No
5	Increased pan-drug resistance by extracellular membrane protective barriers	Yes	No	No	No
6	Bi-directional oncocell translocation between neighboring or long-distance tumors via CCT networks	Yes	No	No	No
7	Increased survival ability by elevated nutrient taking capacities of integrated primary tumor network systems	Yes	No	No	No
8	Increased survival ability by by elevated nutrient taking capacities of integrated secondary tumor network systems	Yes	No	No	No
9	Increased survival ability by by elevated nutrient taking capacities of integrated primary and secondary tumor networks	Yes	No	No	No
10	Ecellulation, auto-entry and alloentry provide membrane-enclosed protected environments for cellular activities	Yes	No	No	No
11	Protected oncocell dissemination in membrane-enclosed tube-shaped freeway systems	Yes	No	No	No
12	Cancer bone metastasis via CCT invasion into hard tissue	Yes	No	No	No
13	Cancer brain metastasis via CCT invasion through blood-brain barrier	Yes	No	No	No
14	Wrapping tumors with multiple and many CCT protection layers	Yes	No	No	No
15	Form CCT superstructures in cancer tissues for massive CCT invasion in tissues with heterogeneous densities and textures	Yes	No	No	No
16	Harbor many CCTs and oncocells in normal tissues adjacent tumor tissues (NAT)	Yes	No	No	No
17	Regeneration of CC membranes and CCTs in multiple times	Yes	No	No	No
18	Generate large 3D networks	Yes	No	No	No
19	Upon chemotherapy, tumor cells are dispersed into other sites in membrane-enclosed systems followed by tumor relapse and cancer survival	Yes	No	No	No
20	Generate extracellular bio-membrane systems beyond circulation systems and present immune attack/therapy cold	Yes	No	No	No
21	Dynamic extracellular biomembrane systems protect oncocell activities and behaviors inside	Yes	No	No	No
22	Extracellular biomembrane system generation, degradation and regeneration protect tumor progression and evolution	Yes	No	No	No
23	Additional membrane barrier decreases efficiency of nutrient molecule taking and metabolic waste molecule diffusion	Yes	No	No	No
24	Additional membrane generation increases energy consuming	Yes	No	No	No
25	Introduce tissue structure interference and damage and biological function failure into normal tissues	Yes			Yes

Fig. S18. Advantages and disadvantages of CC/CCT, CT and CTNS in comparison to other organelles, compartments and cellular activities beyond CC/CCT *in vivo*.

VIDEO LEGENDS:

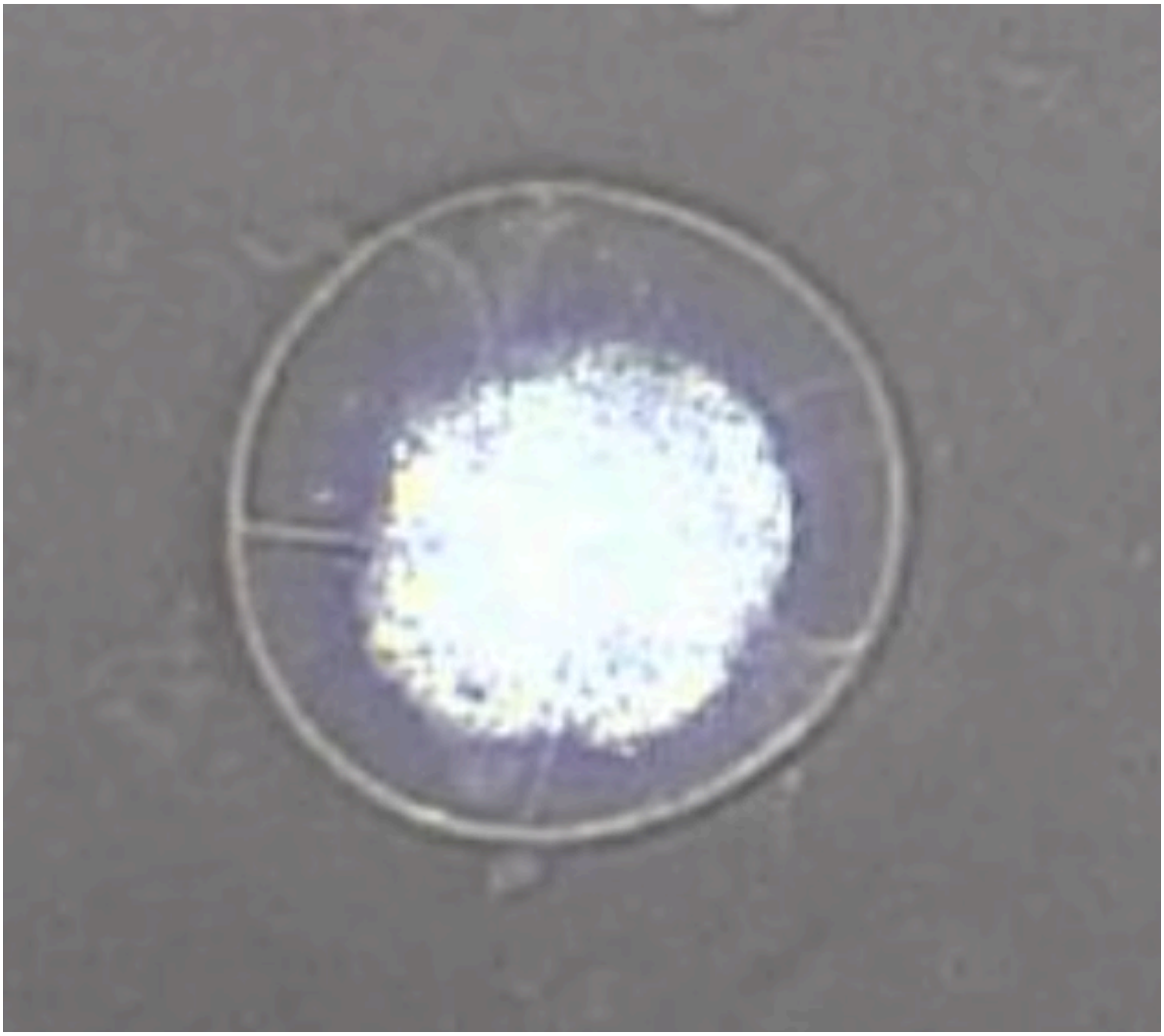

Movie S1. A Bxpc3 pancreas cytocapsular tumorsphere generates a large and spherical cytocapsula enveloping the tumorsphere. The tumorsphere engender large quantities of cytocapsulasomes (membrane-enclosed tiny particles in white color in the movie) covering the surfaces of itself. Cytocapsulasomes can detach and move into the cytocapsular lumen fluids in a random mobility format. Cytocapsulasomes can reach the enlarged cytocapsula membrane, and integrate into cytocapsula membrane, and increase the membrane area sizes of cytocapsula membranes, driving cytocapsula growth or cytocapsular tube elongation. Representative bright field microscope images are extracted and shown in **Fig. S6**.

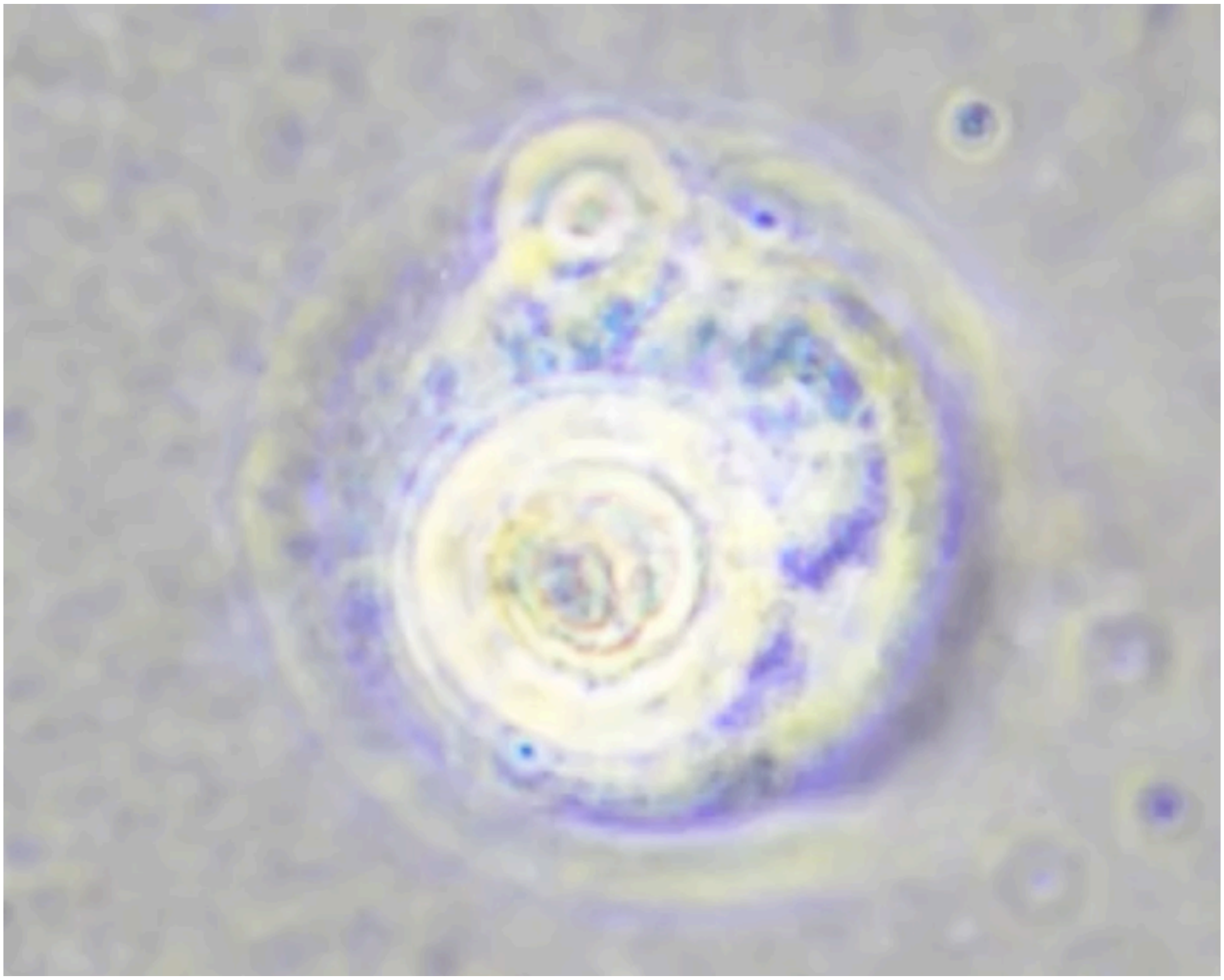

Movie S2. A Bxpc3 pancreas cytocapsular tumorsphere generates a large and spherical cytocapsula enclosing the tumorsphere, and engender a short and big “L” shaped CCT inside the CC lumen. Two individual cytocapsular oncocell in the CC lumen generate their independent CCs and perform ecellulation, and leave two acellular CCs in the large CC lumen. The cytocapsular tumorsphere generates a lot of cytocapsulasomes, and many of them are detached, migrate and arrive at, and contact with the inner side of the enlarged CC membrane. Cytocapsular oncocell engendered cytocapsulasomes are building blocks and shuttering cargos for CC growth and CCT elongation. Representative bright field microscope images are extracted and shown in **Fig. S7**.

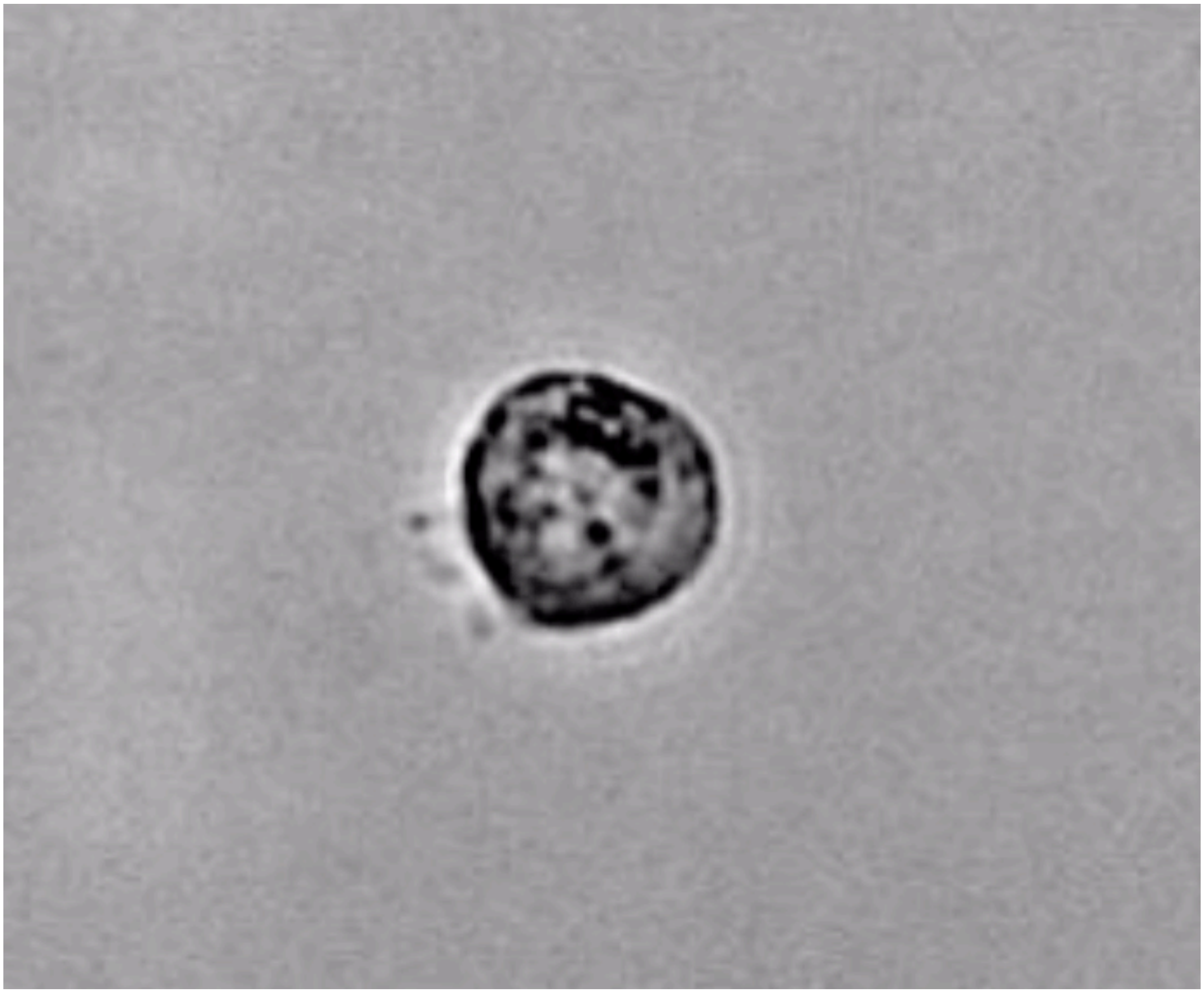

Movie S3. Single breast MCF-7 cancer cell in CC/CCT culture kit matrix generates a cytocapsula enclosing itself, and produces a cytocapsular oncocell. The cytocapsular oncocell employs bleb-based mobility for migration forward, and engender a cytocapsular tube (CCT) behind. Subsequently, the cell switch into a lamellipodia-based motility and migrate in the CCT it generated and migrate back. Single cytocapsular oncocells can generate CCT and migrate inside. Representative DIC images are extracted and shown in **Fig. S9A**.

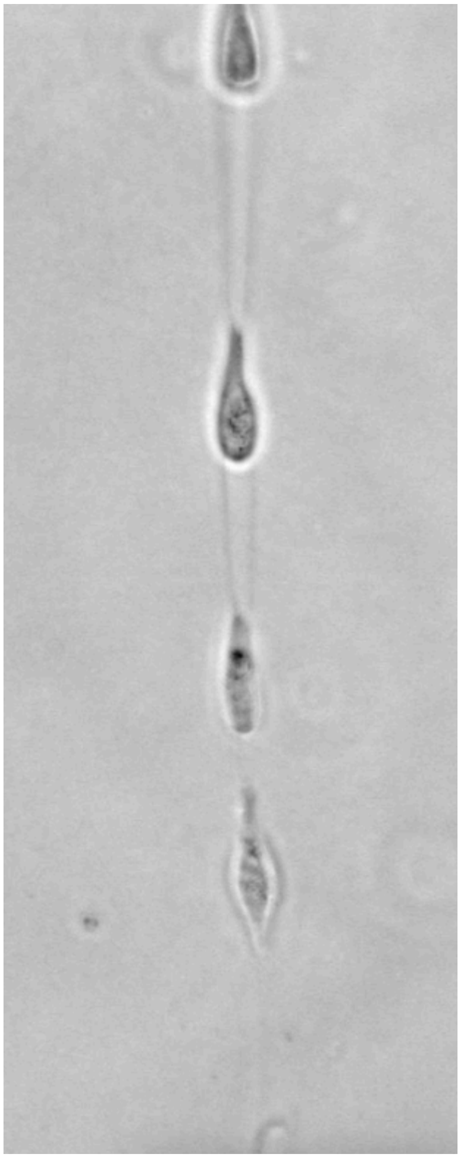

Movie S4. Multiple cytocapsular oncocells in a single CCT employ a single epithelial migration format, and dynamically and bi-directionally migrate in the CCT. The polarized, thin and long cytocapsular oncocells in the CCT migrate forward with periodic protrusion and retraction of the leading lamellipodia and movement of the blunt cell rear. During cancer cell migration in CCT, CCT membranes tightly adhere to oncocell cytoplasm membranes and dynamically increase/decrease the CCT diameter/width locally, displaying considerable CCT membrane elasticity. The polarized and mesenchymal cytocapsular oncocells in CCTs can freely switch the migration direction back and forth and can thus migrate bi-directionally in CCTs. Representative DIC images are extracted and shown in **Fig. S9F**.

SI References:

1. Gerstberger S, et al (2023) Metastasis. *Cell*. **186**, 564-1579 (2023).
2. Hebert D, et al (2023) Dissecting metastasis using preclinical models and methods. *Nat Rev Cancer*. **23**, 391-407.
3. FDA(2020) https://www.accessdata.fda.gov/scripts/cdrh/cfdocs/cfRL/rl.cfm?lid=740305&lpcd=MVU
4. Lawrence R, et al (2023) Circulating tumour cells for early detection of clinically relevant cancer. *Nat Rev Clin Oncol*. **20**, 487-500.
5. Nolan E, et al (2023) Deciphering breast cancer: from biology to the clinic. *Cell*. **186**, 708-1728.
6. Peters A, et al (2016) The calcium pump plasma membrane Ca(2+)-ATPase 2 (PMCA2) regulates breast cancer cell proliferation and sensitivity to doxorubicin. Sci Rep. **6**, 25505.
7. Everley A, et al, Quantitative cancer proteomics: stable isotope labeling with amino acids in cell culture (SILAC) as a tool for prostate cancer research. *Mol Cell Proteomics*. **3**, 729-35 (2004).
8. Yi T, et al, Quantitative phosphoproteomic analysis reveals system-wide signaling pathways downstream of SDF-1/CXCR4 in breast cancer stem cells. *Proc Natl Acad Sci USA*. **111,** E2182-90 (2014).
